# External validation and application of a machine learning–based model for diabetes progression in prediabetes

**DOI:** 10.3389/fendo.2026.1746570

**Published:** 2026-03-23

**Authors:** Song Wang, Qi Huang, Yuxuan Luo, Yingying Luo, Honghan Wu, Zhouhui Lian, Linong Ji, Xiantong Zou

**Affiliations:** 1 Department of Endocrinology and Metabolism, Beijing Key Laboratory of Innovative Drug and Device Translation in Endocrine and Metabolic Diseases, Peking University People's Hospital, Beijing, China; 2 Wangxuan Institute of Computer Technology, Peking University, Beijing, China; 3 Peking University Diabetes Center, Beijing, China; 4 School of Health and Wellbeing, University of Glasgow, Glasgow, United Kingdom

**Keywords:** intervention, machine learning, prediabetes, prediction models, risk stratification

## Abstract

**Introduction:**

This study externally validated a machine learning–based model for type 2 diabetes progression (ML-PR) and evaluated its clinical utility in individuals with prediabetes.

**Methods:**

We included 3,081 participants from the Diabetes Prevention Program (DPP) and the DPP Outcome Study (DPPOS). The ML-PR model was assessed using dicrimination, calibration curves, and decision curve analysis, and its performance was compared with existing diabetes prediction models. Based on ML-PR scores, patients were stratified into high- or low-risk categories. Cox proportional hazards and logistic regression models were used to evaluate the incidence of type 2 diabetes, microvascular complications, and cardiovascular events across risk and intervention groups.

**Results:**

The ML-PR model achieved an area under the ROC curve of 0.74 (95% confidence interval: 0.71–0.78) for predicting 3-year progression to type 2 diabetes. Calibration and decision curve analyses indicated good agreement and net clinical benefit. High-risk individuals exhibited a significantly higher risk of developing type 2 diabetes in both the DPP and DPPOS cohorts (P < 0.001), as well as a 67% increased risk of microvascular complications in DPPOS (P < 0.001), though no significant difference in cardiovascular risk was observed. Significant interactions between treatment and risk group were identified, indicating that high-risk participants benefited more from lifestyle modification and metformin interventions (P for interaction = 0.03 in DPP; P = 0.014 in DPPOS).

**Discussion:**

Externally validated in U.S. cohorts, the ML-PR model effectively identifies individuals with prediabetes at elevated risk of diabetes progressing and microvascular complications. These findings suggest that intensive lifestyle interventions and metformin therapy may be particularly beneficial for individuals at higher risk, highlighting the potential for more precise treatment strategies in type 2 diabetes.

## 1 Introduction

Prediabetes represents an intermediate state between normal glucose regulation and type 2 diabetes, including impaired fasting glucose, impaired glucose tolerance, and elevated HbA1c (1). In 2021, 9.1% and 5.8% of the global population had impaired glucose tolerance and impaired fasting glucose, respectively, with these figures projected to increase, highlighting the increasing burden of metabolic diseases (2). Individuals with prediabetes face a substantially elevated risk of developing type 2 diabetes (3). The China Da Qing Diabetes Prevention Study revealed that, without intervention, 65.8% of prediabetic individuals developed type 2 diabetes within 6 years, with this rate increasing to 92.8% after 20 years of follow-up (4). In addition, prediabetes is also associated with an increased risk of cardiovascular disease (CVD) and microvascular events, largely attributable to elevated glucose levels and the subsequent onset of type 2 diabetes (5). Therefore, early intervention in prediabetic individuals is critical for controlling type 2 diabetes progression.

Studies have demonstrated that lifestyle interventions and pharmacological treatments can be effective for preventing the progression of prediabetes to type 2 diabetes (5–7). However, the rate and outcomes of progression from prediabetes to diabetes vary widely among individuals. Approximately 5% to 10% of prediabetic individuals progress annually to type 2 diabetes, whereas a similar proportion revert to normal glucose regulation (8). Furthermore, therapeutic responses to interventions in individuals with prediabetes show considerable heterogeneity. The Diabetes Prevention Program (DPP) demonstrated limited efficacy of lifestyle interventions in participants with fasting plasma glucose ≥110 mg/dL, body mass index ≥35 kg/m^2^, and age<45 years (7).

Recently, machine learning approaches have gained attention because of their ability to handle complex data, integrate diverse variables, and enhance predictive accuracy. We previously developed a machine learning–based model for type 2 diabetes progression(ML-PR) using XGBoost in a Chinese prediabetes cohort, achieving favourable discrimination and calibration (9). The ML-PR model incorporates five variables: fasting plasma glucose (FPG), 2-hour postprandial glucose after a 75 g glucose load (PG2h), HbA1c, high-density lipoprotein cholesterol (HDL-C), and triglycerides (TG). The ML-PR accurately predicted 1-year diabetes progression externally in the Beijing Prediabetes Reversion Program, with an area under the receiver operating characteristic curve (ROC AUC) of 0.80 (95% CI: 0.74–0.86). Stratifying patients by ML-PR risk levels revealed differential treatment effects of pioglitazone for diabetes prevention among prediabetic individuals (9). Despite these valuable insights, the ML-PR has limitations, including a short prediction period and validation in only monoethnic (Chinese) cohorts. This study aims to externally validate the ML-PR in a cohort of prediabetic individuals across the United States, facilitating patient stratification and identification of treatment response heterogeneity.

## 2 Materials and methods

### 2.1 Study data and participants

Data from two randomized clinical trials, the Diabetes Prevention Program (DPP, NCT00038727) and the Diabetes Prevention Program Outcome Study (DPPOS, NCT03441750), were utilized for *post hoc* analysis. All participants from these trials who met the intention-to-treat analysis criteria were included. Data from DPP and DPPOS were obtained through the National Institute of Diabetes and Digestive and Kidney Diseases. This study received ethical approval from the Peking University People’s Hospital Ethics Committee (Approval No. 2024PHB050-001).

#### 2.1.1 DPP study

The DPP clinical trial (1996–2002) included prediabetes participants across the United States, all of whom met the WHO 1985 criteria for impaired glucose tolerance with fasting plasma glucose levels between 5.3 and 6.9 mmol/L and without prior cardiovascular disease(CVD), poorly controlled hypertension, severe liver dysfunction, or severe renal disease. The participants were randomly assigned to one of three intervention groups: a standard lifestyle plus placebo group(placebo group), a standard lifestyle plus metformin group(metformin group), and an intensive lifestyle group (lifestyle group). The two primary goals of the lifestyle intervention were at least a 7% reduction in body weight and at least 150 minutes per week​of physical activity at brisk-walking-like intensity. A planned fourth intervention group, consisting of troglitazone plus a standard lifestyle, was discontinued due to drug-induced liver toxicity. Type 2 diabetes is diagnosed annually via an oral glucose tolerance test or semiannually by fasting blood glucose measurements (7).

#### 2.1.2 DPPOS study

The DPPOS (2002–2019) was an extension of the DPP that followed the original participants to assess the long-term impact of the initial interventions on type 2 diabetes and related complications, including microvascular and cardiovascular outcomes. Importantly, no new randomization was conducted in DPPOS, and participants retained their original DPP intervention assignment. During DPPOS, all participants were provided with lifestyle maintenance courses, including weight management and physical activity, while the original lifestyle group received additional biannual supplemental courses to reinforce behavioural self-management, and the metformin group continued their medication. Demographic data and laboratory measurements were collected as previously described (10).

### 2.2 Outcomes

The primary outcome of this study was the progression rate from prediabetes to type 2 diabetes. The secondary outcomes included the incidence of CVD events and microvascular events.

Type 2 diabetes was diagnosed using the World Health Organization 1999 criteria: FPG≥7.0 mmol/L and/or PG2h ≥11.1 mmol/L. CVD events were defined as major adverse cardiovascular events (MACEs), including nonfatal myocardial infarction, nonfatal stroke, or fatal cardiovascular disease. Microvascular events included nephropathy (defined as two consecutive urine albumin–creatinine ratios≥30 mg/g, estimated glomerular filtration rate<45 ml/min/1.73 m^2^, or renal failure), retinopathy (diagnosed by the early treatment diabetic retinopathy study grade≥20 or treated with laser/vitrectomy) (11), and neuropathy (assessed by loss of protective sensation via the 10-gram monofilament test).

### 2.3 Introduction of models

The ML-PR model was developed using the Extreme Gradient Boosting (XGBoost) algorithm implemented via the caret framework for binary classification. Five predefined clinical predictors(FPG, PG2h, HbA1c, HDL-C, and TG) were included, with continuous variables retained on their original scale. Model training was optimized for ROC using repeated 5-fold cross-validation. Hyperparameters were prespecified to emphasize generalizability (270 boosting iterations, maximum tree depth of 3, learning rate of 0.01, column subsampling rate of 0.6, minimum child weight of 3).ML-PR successfully predicted the 1-year progression in the Beijing Prediabetes Reversion Program (n=1936), with ROC AUC of 0.80 (95%CI:0.74-0.86). The tertiles defined by the ML-PR model score were used for patient stratification in the Beijing Prediabetes Reversion Program, with a cut-off value of 0.6 distinguishing the high-risk group from the non-high-risk groups. The score of ML-PR was calculated via the R package or webpage (http://models.ourboat.cn:9001/index?type=1, with access granted upon reasonable request and authorization by the research team).To enhance the interpretability of the ML-PR model, we performed Shapley Additive exPlanations (SHAP) analysis in the external validation cohort. SHAP values were computed to quantify the marginal contribution of each predictor to individual risk predictions. As XGBoost natively handles missing values by learning optimal default split directions during tree construction, explicit imputation and data reduction were not used during model validation. To assess calibration in the DPP population, we performed a recalibration analysis using the placebo group by fitting a Cox proportional hazards model with the ML-PR score as the sole predictor to estimate recalibration parameters, including both calibration slope and intercept. The Diabetes Prediction Model(DPM) was developed using proportional hazards regression to predict risk for progression to diabetes in the DPP (12), and Framingham score was developed through regression models from Framingham Offspring Study to predict new T2D (13).PREVENT(Predicting Risk of CVD Events), developed and validated in large European cohorts, is a risk prediction tool that estimates long-term cardiovascular disease risk by integrating traditional, lifestyle, and metabolic factors.

The equation of the Diabetes Prediction Model score (12) is


Risk=1.8(FPG[mg/dl]−90)+3.33(HbA1c[%]−3)+13(History of high blood glucose)+0.055(TG[mg/dl])+0.3(WC[cm]−60)+0.4(200−Height[cm])+43.3(Waist to hip ratio−0.5)

The equation of the Framingham score (13) is


$$\begin{array}{l} Risk=10*I\left(6.1\leq FPG\left[mmol/l\right]<7.0\right)+2*I\left(25\leq BMI<30\right)+5*I(BMI\geq 30)+2*I\left(SBP\left[mmHg\right]\geq 130\right)+2*I(SBP\left[mmHg\right]<130\&DBP\left[mmHg\right]\geq 85)+3*I\left(Male\&HDL-C\left[mmol/L\right]<1.03\right)+3*I(Female\&HDL-C\left[mmol/L\right]<1.29) \end{array}$$

### 2.4 Data analysis

ML-PR scores were derived from participants’ baseline variables and subsequently applied to the analysis of data from both the DPP and DPPOS cohorts. Given the known effects of lifestyle and metformin interventions, the placebo arm of the DPP was selected as the external control cohort, because it received standardized education but no active pharmacological intervention. Model performance for predicting 3-year type 2 diabetes progression was externally validated via the area under the receiver operating characteristic curve (ROC AUC), calibration curves, and decision curve analysis. Additionally, ROC AUCs for type 2 diabetes progression prediction were compared with those from conventional risk assessment tools, including DPM (12) and Framingham score (13). Receiver operating characteristic curves were compared via DeLong’s test. A cutoff score of 0.6 from the Chinese validation study was used in the DPP to separate participants into high- and low-risk groups. Those scoring above or equal to 0.6 were considered high-risk, and those below 0.6 low-risk. Alternative ML-PR cutoff values were evaluated using tertile- and quartile-based thresholds. In addition, an optimal cutoff was derived in the DPP cohort using the Youden index based on 3-year incident type 2 diabetes, defined as the ML-PR value that maximized sensitivity + specificity − 1.

Risk stratification and treatment heterogeneity tests were conducted between risk groups according to the ML-PR score. Cox proportional hazards regression was used to estimate the absolute risk differences in the incidence of type 2 diabetes and CVD across groups stratified by risk level. Additionally, Cox models were used to assess how interventions influenced type 2 diabetes progression and CVD incidence in both the high- and low-risk groups. Interaction tests were conducted to evaluate whether treatment effects differed significantly between risk groups, with interaction P values derived from the Wald test. To illustrate the potential clinical implications of ML-PR–guided risk stratification, subgroup-specific numbers needed to treat (NNT) were estimated in the DPP cohort. NNT values were calculated separately for the low- and high-risk groups as the inverse of the absolute risk reduction between placebo and intervention groups over 3 years. Because the precise timing of microvascular events was unavailable, logistic regression analysis was employed to compare the frequency of microvascular events among risk groups and to test for differences in treatment effects by risk group.

We performed sensitivity analyses to test the robustness of our results by (1): using tertiles and quartiles of the ML-PR score as alternative cut-offs (2); stratifying participants with the DPM model using quartiles as cut-off scores; and (3) applying the PREVENT model to define CVD risk groups. Prediabetes patients with a score of PREVENT ≥7.5% were defined as the high-CVD risk group (14).

For normally distributed continuous variables, the data are reported as the means ± standard deviations. Baseline continuous variables were compared across two risk groups via an independent samplest-test (for homogeneity of variance) or the Mann–Whitney U test (for nonhomogeneous variance). For categorical variables, the data are reported as N (%). Baseline categorical variables were compared via the chi-square test or Fisher’s exact test.

The datasets generated in the current study are available from the National Institute of Diabetes and Digestive and Kidney Diseases. Statistical analyses were conducted via SPSS Statistics 27 and R version 4.3.3(rms, survival, timeROC, ggDCA, and the forestploter package).

## 3 Results

### 3.1 Model performance

**Figure 1:**
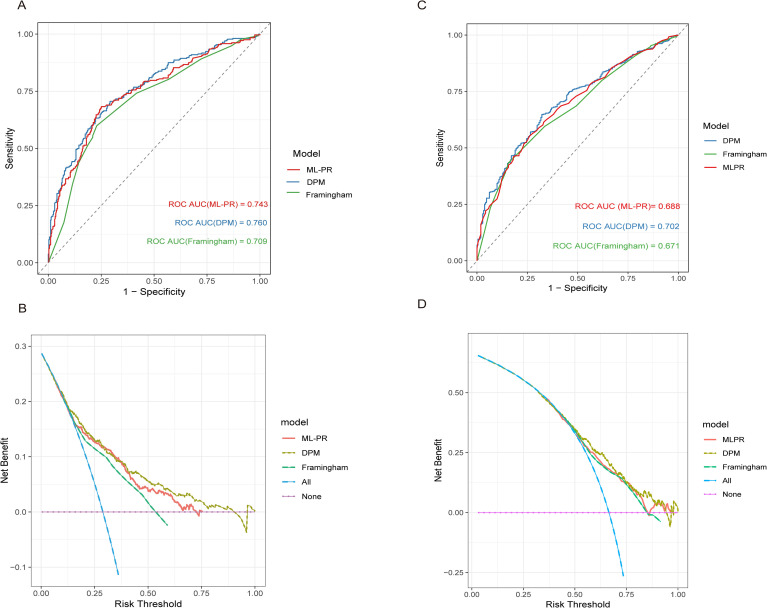
Performance of models among prediabetic individuals receiving placebo in DPP and DPPOS cohort. **(A)** Receiver operating characteristic curves for the machine learning-based diabetes progression model (ML-PR) (red), diabetes prediction model (DPM, blue) and Framingham score (green) in predicting 3-year type 2 diabetes progression in the DPP placebo arm (n=1030). **(B)** Decision curve analysis of the ML-PR model (red), DPM (dark green) and Framingham score (green) in the DPP placebo arm (n=1030). Purple line: Assuming that no patient progressed to type 2 diabetes within 3 years. Blue line: Assuming that all patients progressed to diabetes within 3 years. **(C)** Receiver operating characteristic curves for the machine learning-based diabetes progression model (ML-PR) (red), diabetes prediction model (DPM, blue) and Framingham score (green) in predicting 21-year type 2 diabetes progression in the DPPOS placebo arm (n=782). **(D)** Decision curve analysis of the ML-PR model (red), DPM (dark green) and Framingham score (green) in the DPPOS placebo arm (n=782). Purple line: Assuming that no patient progressed to type 2 diabetes within 3 years. Blue line: Assuming that all patients progressed to diabetes within 3 years. DPP, Diabetes Prevention Program; DPPOS, Diabetes Prevention Program Outcome Study; DPM, diabetes prediction model; ML-PR, machine learning–based model for type 2 diabetes progression; ROC AUC, area under the curve of the receiver operating characteristic curve.

The DPP cohort comprised 3,081 prediabetes participants: 1,024 in the lifestyle group, 1,027 in the metformin group, and 1,030 in the placebo group. The DPPOS reported 21-year follow-up data among participants: 2306 for diabetes-related outcomes, 2130 for microvascular complications, and 3055 for CVD outcomes. The distribution of ML-PR score by 0.2 increments in the DPP cohort was described in **Supplementary Figure 1**. In the placebo group, the ML-PR model predicted 3-year type 2 diabetes risk, with an ROC AUC of 0.74 (95% confidence interval[CI]: 0.71–0.78). The DPM, which was originally developed using the DPP cohort, achieved an ROC AUC of 0.76 (95% CI: 0.73–0.80) in the placebo group, while the Framingham model yielded an ROC AUC of 0.71 (95% CI: 0.67–0.74). The ML-PR model outperformed the Framingham model (*P* = 0.042) and showed a similar ROC AUC to the DPM model (*P* = 0.15) (**Figure 1A**). The calibration curves demonstrated strong concordance between the predicted and observed 3-year type 2 diabetes progression risks (**Supplementary Figure 2A**). Additionally, decision curve analysis (DCA) was used to evaluate the clinical utility of the ML-PR model for guiding management decisions in patients at risk for 3-year type 2 diabetes (**Figure 1B**).In the SHAP analysis, FPG and PG2h contributed the most to the model, with HbA1c and lipid-related indicators ranking next in importance (**Supplementary Figure 3**).When the ML-PR score was recalibrated using the DPP placebo group, discrimination remained largely unchanged compared with the original ML-PR score(**Supplementary Figure 4**). The ML-PR model, applied to the DPPOS cohort, yielded a 21-year type 2 diabetes incidence prediction with an ROC AUC of 0.69 (95% CI: 0.64–0.72) in the placebo group (**Figure 1C**). No statistically significant differences were observed among the ROC AUCs of the ML-PR, DPM, and Framingham models in the DPPOS cohort (**Supplementary Table 1**, ML-PR vs. DPM: *P* = 0.11; ML-PR vs. Framingham: *P* = 0.78; DPM vs. Framingham: *P* = 0.051). DCA was performed to evaluate the clinical utility of the ML-PR, DPM, and Framingham models in predicting 21-year type 2 diabetes incidence within the DPPOS cohort (**Figure 1D**). 1984 participants were categorized into the low-risk group, and 1097 participants were categorized into the high-risk group. We observed that age, blood pressure, weight, body mass index, waist circumference, HbA1c, FPG, PG2h,insulin resistance, total cholesterol, HDL-C, low-density lipoprotein cholesterol, alanine transaminase, aspartate transaminase, and serum creatinine increased with increasing type 2 diabetes progression risk. However, other cardiovascular risk factors, including triglycerides and the urinary albumin/creatinine ratio, were similar between the subgroups. The distribution of intervention types was similar across risk groups (**Table 1**).

**Table 1:** Baseline clinical characteristics of different risk groups.

Characteristics	Low-risk group(n=1984)	High-risk group(n=1097)	*P value*
Range of ML-PR score	0.12-0.60	0.60-0.91	
Sex, n (%)			<.001
Male	599 (30.2)	429 (39.1)	
Female	1385 (69.8)	668 (60.9)	
Systolic blood pressure (mmHg)	123.1 ± 14.5	126.1 ± 14.9	<.001
Diastolic blood pressure (mmHg)	78.2 ± 9.1	79.0 ± 9.6	0.013
History of high cholesterol,n(%)	690(34.8)	420(38.3)	0.055
History of hypertension, n(%)	501(25.3)	334(30.4)	0.002
Age (year)	50.4 ± 10.5	51.7 ± 10.8	0.002
Body mass index (kg/m^2^)	33.4 ± 6.3	35.1 ± 7.3	<.001
Weight (kg)	92.3 ± 19.1	98.8 ± 21.8	<.001
Waist circumference (cm)	103.3 ± 14.0	108.0 ± 15.1	<.001
Fasting Insulin (uU/mL)	24.9 ± 13.8	29.6 ± 16.5	<.001
30 Minute Insulin (uU/mL)	98.7 ± 61.7	98.4 ± 63.6	0.885
Fasting Proinsulin (pM)	15.2 ± 10.7	23.4 ± 17.2	<.001
FPG (mg/dL)	103.8 ± 4.8	113.4 ± 8.2	<.001
PG2h(mg/dL)	160.4 ± 16.4	172.3 ± 15.3	<.001
HbA1c (%)	5.7 ± 0.4	6.3 ± 0.5	0.001
Triglycerides (mg/dL)	162.2 ± 95.7	164.0 ± 89.1	0.603
Cholesterol (mg/dL)	202.9 ± 35.0	205.8 ± 37.8	0.033
HDL-C(mg/dL)	46.9 ± 12.2	43.4 ± 10.8	<.001
LDL-C (mg/dL)	123.4 ± 31.8	129.3 ± 34.0	<.001
UACR(mg/g)	12.4 ± 40.2	15.6 ± 47.3	0.06
Alanine transaminase(U/L)	19.8 ± 12.5	21.3 ± 12.2	0.001
Aspartate transaminase (U/L)	20.2 ± 7.8	21.3 ± 10.2	0.001
Serum creatinine (mg/dl)	0.77 ± 0.17	0.81 ± 0.18	<.001
Assign, n (%)			0.900
Placebo	658(33.2)	372(33.9)	
Lifestyle	660(33.3)	364(33.2)	
Metformin	666(33.5)	361(32.9)	
Incidence of T2DM, n (%)	241(7.8)	414(35.6)	<.001

For normally distributed continuous variables, the data are reported as the means ± standard deviations (SDs). Baseline continuous variables were compared across two risk groups via an independent samples ttest (for homogeneity of variance) or the Mann–Whitney U test (for nonhomogeneous variance). For categorical variables, the data are reported as N (%). Baseline categorical variables were compared via the chi-square test or Fisher’s exact test.

FPG, fasting plasma glucose; HDL-C, high-density lipoprotein cholesterol; LDL-C, low-density lipoprotein cholesterol; ML-PR, machine learning–based model for type 2 diabetes progression; PG2h, 2-hour postprandial plasma glucose after 75 g glucose administration; T2DM, type 2 diabetes mellitus; UACR, urinary albumin/creatinine ratio.

### 3.2 Risk stratification

**Figure 2:**
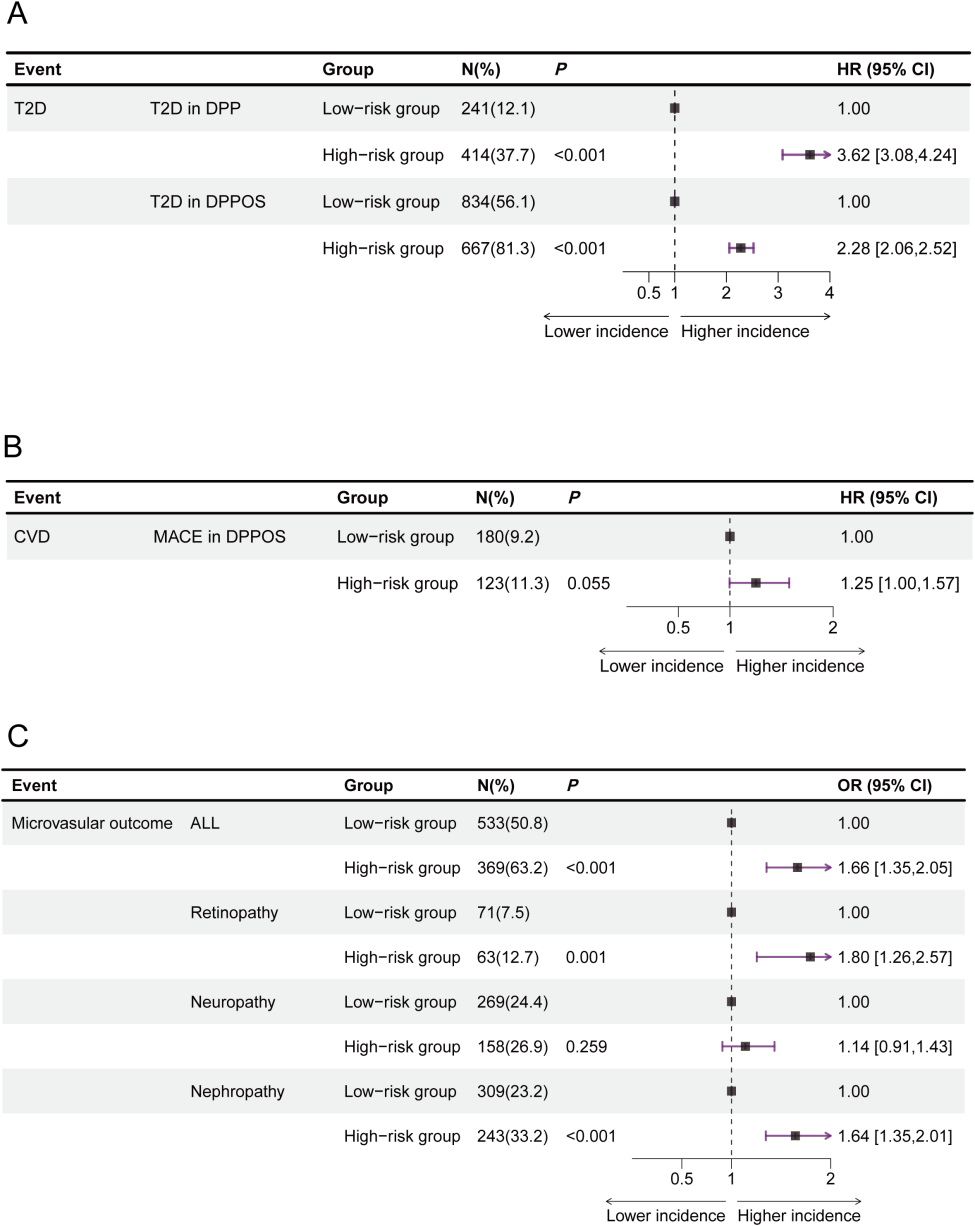
Estimated risks of type 2 diabetes, cardiovascular disease (CVD), and microvascular outcomes across different risk groups. HR for incident type 2 diabetes **(A)** and CVD **(B)** were calculated viaCox proportional hazards regression, accounting for time-to-event data. Ors for microvascular outcomes **(C)** were derived from logistic regression due to the absence of recorded onset times for these outcomes. All estimates are presented with 95% CIs. The data are summarized as N (%) for categorical representation. CVD, cardiovascular disease; CI, confidence interval; DPP, Diabetes Prevention Program; DPPOS, Diabetes Prevention Program Outcome Study; HR, hazard ratio; MACE, major adverse cardiovascular events; OR, odds ratio; T2D, type 2 diabetes.

The 3-year incidence of type 2 diabetes differed significantly between the high- and low-risk groups (*P* < 0.001) in the DPP study. In the longitudinal DPPOS follow-up, 56.1% of participants in the low-risk group and 81.3% in the high-risk group developed type 2 diabetes. Cox regression analysis revealed that the 21-year incidence of type 2 diabetes was 128%greater in the high-risk group than in the low-risk group (81.3% versus 56.1%, hazard ratio[HR]=2.28 [95%CI:2.06,2.53], *P* < 0.001, **Figure 2A**). Additionally, the 21-year risk of microvascular outcome increased by 67% in the high-risk group (63.2% versus 50.8%, OR = 1.67 [95%CI:1.35,2.05], *P* < 0.001, **Figure 2C**). Subgroup analyses revealed that differences in microvascular outcome were primarily due to increased nephropathy and retinopathy rather than neuropathy (**Figure 2C**). However, there was no significant difference in CVD incidence between the different risk groups (11.3% versus 9.2%, HR = 1.25[95%CI:0.99,1.58], *P* = 0.055, **Figure 2B**).

### 3.3 Intervention strategies for different risk groups

**Figure 3:**
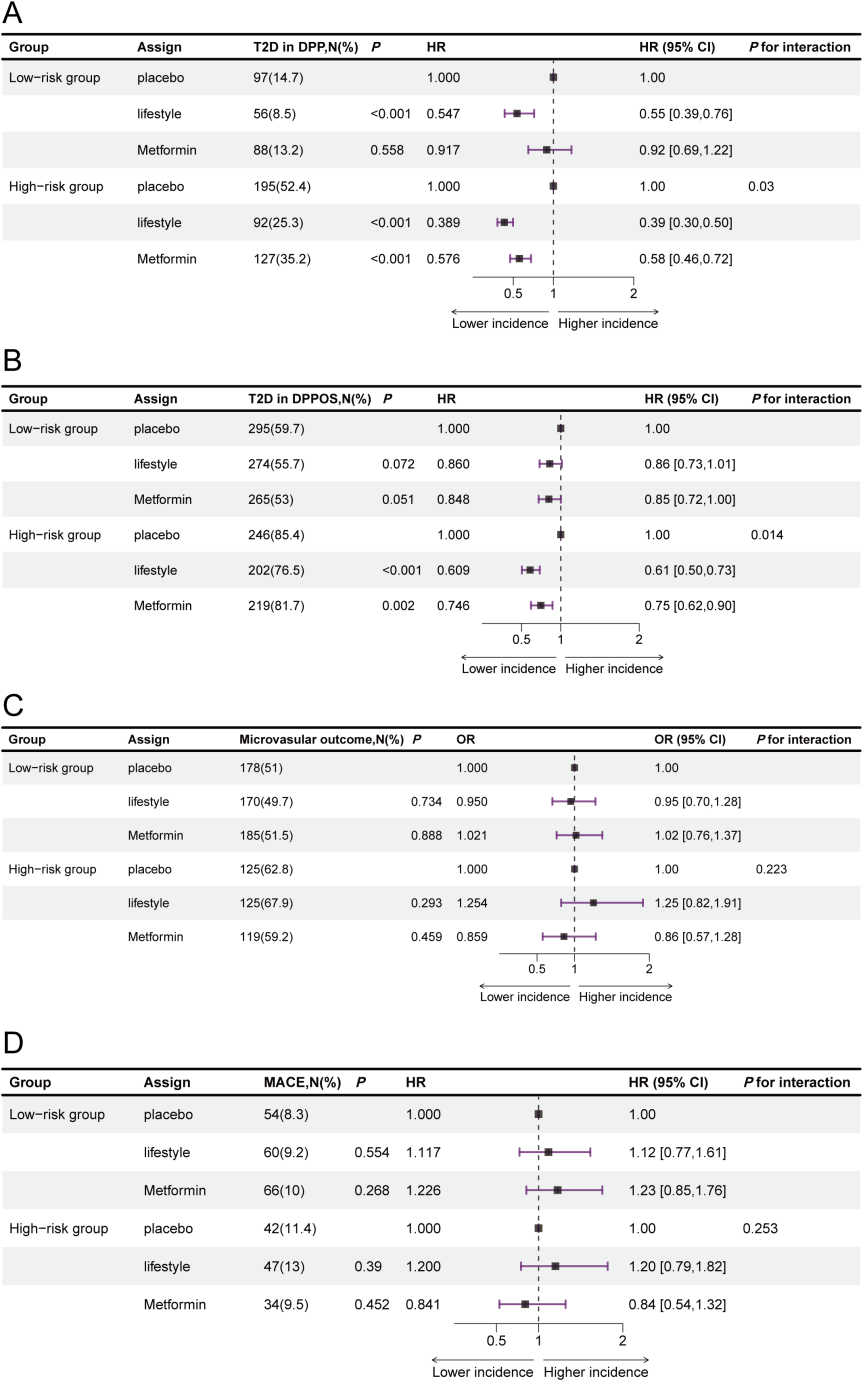
Type 2 diabetes progression, cardiovascular events and microvascular outcomes under interventions across risk groups. HR in reference to placebo intervention for progression to type 2 diabetes in the high- and low-risk groups at the end of DPP **(A)** and DPPOS **(B)**. HR in reference to placebo intervention for progression to CVD in high- and low-risk groups at the end of DPPOS **(C)**. OR in reference to placebo intervention for progression to microvascular outcomes in the high- and low-risk groups at the end of DPPOS **(D)**. HR for type 2 diabetes and CVD events were analysed via Cox regression, and OR for microvascular outcomes was analysed via logistic regression, as the exact time of onset for microvascular outcomes was not recorded. The P value for interaction was assessed via the Wald test. All estimates are presented with 95% CIs. The data are summarized as N (%) for categorical representation. CVD, cardiovascular disease; CI, confidence interval; DPP, Diabetes Prevention Program; DPPOS, Diabetes Prevention Program Outcome Study; HR, hazard ratio;MACE, major adverse cardiovascular events; OR, odds ratio; T2D, type 2 diabetes.

**Figure 4:**
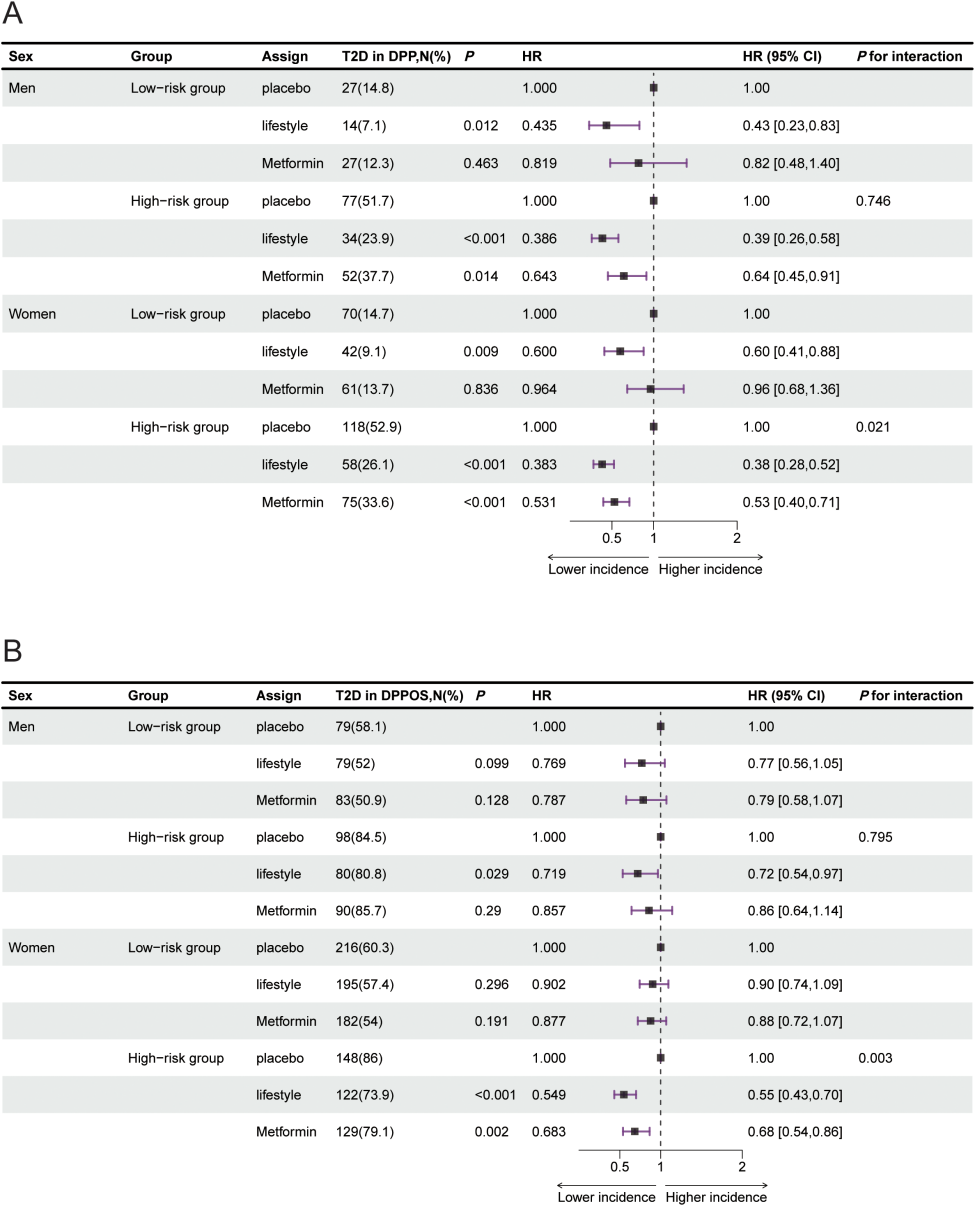
Type 2 diabetes progression under interventions across risk groups in men and women respectively. HR in reference to placebo intervention for progression to type 2 diabetes in the high- and low-risk groups in men and women at the end of DPP **(A)** and DPPOS **(B)**. HR for type 2 diabetes were analysed via Cox regression. The P value for interaction was assessed via the Wald test. All estimates are presented with 95% CIs. The data are summarized as N (%) for categorical representation. CVD, cardiovascular disease; CI, confidence interval; DPP, Diabetes Prevention Program; DPPOS, Diabetes Prevention Program Outcome Study; HR, hazard ratio; MACE, major adverse cardiovascular events; OR, odds ratio; T2D, type 2 diabetes.

In the DPP and DPPOS cohorts, significant interaction effects were observed between risk groups and interventions concerning long-term type 2 diabetes progression (*P* for interaction was 0.03 for DPP and 0.014for DPPOS) (**Figures 3A, B**). In the DPP, metformin had no significant effect on reducing the risk of type 2 diabetes progression in low-risk participants, but it significantly reduced the risk by 42% in high-risk individuals (HR = 0.58, 95% CI: 0.46–0.72, *P* < 0.001) (**Figure 3A**). These differences translated into markedly different numbers needed to treat. In the high-risk group, the NNT was approximately 4 for intensive lifestyle intervention and 6 for metformin treatment. In contrast, in the low-risk group, the NNT was approximately 16 for lifestyle intervention and 67 for metformin treatment. In the DPPOS cohort, lifestyle intervention did not significantly reduce the degree of type 2 diabetes progression risk in the low-risk group (HR = 0.86, 95% CI: 0.73–1.01, *P* = 0.072) but significantly reduced the degree of progression risk by 39% in the high-risk group (HR = 0.61, 95% CI: 0.51–0.73, *P* < 0.001) (**Figure 3B**). Under metformin intervention, the reduction in progression risk was more pronounced in the high-risk group (HR = 0.75, 95% CI: 0.62-0.90; *P* < 0.001) compared with the low-risk group in the DPPOS (HR = 0.85, 95% CI: 0.72–1.00; *P* = 0.05) (**Figure 3B**). There were no significant differences among the three interventions in reducing microvascular events (*P* for interaction = 0.223) or CVD events (*P* for interaction = 0.253) in the different risk groups in terms of DPPOS (**Figures 3C, D**). In subgroup analyses, significant interaction effects between risk group stratification and intervention effects on type 2 diabetes progression risk reduction were observed in women, but not in men, within both the DPP and DPPOS cohorts (**Figure 4**).

### 3.4 Sensitivity Analysis

We conducted a complete case analysis with the remaining 3,068 participants, where the models showed a ROC AUC of 0.75 (95% CI: 0.71-0.78) for the placebo group over a 3-year diabetes outcome (n=1024), and an overall AUC of 0.72 (95% CI: 0.70-0.74)(n=3068) (**Supplementary Figure 5**) We analyzed whether the risk stratification and intervention interaction would change if other cut-off values were used. When using the tertile, quartile, or Youden index-based thresholds of ML-PR score in DPP as the cutoff value, the major conclusion was unchanged (**Supplementary Figures 6–S11**). We also stratified participants via DPM (**Supplementary Figure 12**) and assessed the interaction between the intervention and risk groups according to the DPM (**Supplementary Figure 13**). Given that models specifically predicting type 2 diabetes progression showed limited performance in predicting CVD progression among prediabetes patients, the PREVENT model was applied to stratify participants into low- and high-CVD risk groups. The 21-year risks of cardiovascular events and microvascular events were 2.84-fold (*P* < 0.001) and 2.87-fold (*P* < 0.001) greater in the high-CVD risk group than in the low-CVD risk group, respectively (**Supplementary Figure 14**). No significant interaction was observed between intervention type and CVD risk group (**Supplementary Figure 15**). We also assessed the model performance separately among Caucasian, African American, Hispanic, and other racial groups in both DPP and DPPOS. In the DPP and DPPOS cohort, the ML-PR model demonstrated acceptable to good discrimination across Caucasian and African American. Lower AUC estimates were observed in certain subgroups, particularly those with smaller sample sizes (**Supplementary Figure 16**, **Supplementary Table 2**).

## 4 Discussion

In this *post hoc* analysis of the DPP and DPPOS cohorts, we demonstrated the utility of ML-PR in individuals with prediabetes. Originally developed in a Chinese cohort, ML-PR demonstrated transferable, robust predictive performance and effectively identified treatment-response heterogeneity among individuals with prediabetes in a U.S. cohort. Notably, the high-risk subgroup identified by the ML-PR exhibited a significantly elevated risk of diabetes progression and microvascular complications, although the cardiovascular risk remained unaffected. Both lifestyle and metformin interventions were more effective in preventing diabetes progression in this high-risk group.

Our study contributes to the expanding evidence base for predictive models in prediabetes, highlighting the promise of risk stratification tools for type 2 diabetes. Earlier models—including parametric proportional hazards models, LASSO-based algorithms, and machine learning approaches—have shown good internal performance (ROC AUC≥0.7) (15–18). Yet, most models lack robust external validation and long-term predictive data. The Framingham score, which served as a cornerstone prediction tool for estimating 5–10 year diabetes progression risk in U.S. individuals (19), showed limited predictive accuracy when tested in a Chinese prediabetes cohort (9). The DPM model offered performance comparable to the ML-PR but remained confined to internal validation within the DPP with little evidence of generalizability to other settings. By contrast, the ML-PR not only maintained predictive strength but also extended its utility across populations and time horizons. Despite being originally designed for 1-year diabetes progression, it accurately captured 3-year and 20-year risk trajectories in both the DPP and DPPOS cohorts. Beyond discrimination, calibration analyses showed strong alignment between predicted and observed outcomes, with additional metrics (e.g., F1 scores) supporting performance consistency. Decision curve analysis further confirmed clinical utility, with net benefit exceeding default strategies across probability thresholds. Despite being developed in a mono-ethnic Chinese cohort, the ML-PR model demonstrated consistent discriminatory performance across racial groups in the DPP and DPPOS population, supporting its potential cross-racial generalizability, while highlighting the need for further validation in larger and more diverse cohorts. Importantly, the ML-PR achieved these results using only five routinely collected clinical variables, reinforcing its feasibility for real-world application. To improve interpretability, we performed SHAP analysis in the external cohort to quantify the contribution of each predictor to the ML-PR score. FPG and PG2h were the most influential features, followed by HbA1c and lipid parameters. This pattern is biologically plausible, as glycemic markers reflect declining β-cell function and worsening insulin resistance. The greater importance of FPG and PG2h compared with HbA1c likely reflects the DPP cohort characteristics, in which prediabetes was defined using WHO 1985 criteria based on FPG and PG2h. Notably, lipid parameters also contributed meaningfully. Elevated triglycerides and reduced HDL-C are closely linked to insulin resistance. Moreover, studies have identified TG/HDL-C as predictors of diabetes progression (20, 21). The model’s availability online (http://models.ourboat.cn:9001/index?type=1, with access granted upon reasonable request and authorization by the research team) makes it accessible for both clinical and research use, supporting its role as a practical, generalizable tool for precision prediction in prediabetes.

Multiple cutoff values (including tertile-, quartile-, and Youden index–based thresholds) demonstrated comparable performance, supporting the robustness of ML-PR–based risk stratification. Although applying a cutoff derived from the Chinese development cohort to U.S. populations may introduce some degree of population-specific bias, the overall performance remained stable across alternative thresholds. Therefore, the original cutoff of 0.6 was retained for consistency, comparability across cohorts, and ease of clinical implementation. Nonetheless, optimal cutoff selection may vary across populations, and future studies are warranted to explore population-specific thresholds and the potential need for recalibration in different clinical settings.

Recalibration was evaluated to assess model transportability across populations. Adjustment of calibration slope and intercept in the DPP placebo group had minimal impact on discrimination, with AUC values remaining essentially unchanged. Although a population-specific model (MLPR-US) achieved higher performance within the DPP cohort, its reduced discrimination in the Chinese population limited cross-population applicability (Data unshown). Therefore, the original ML-PR model was retained to preserve transportability. Notably, the predictive performance of ML-PR was lower in the DPPOS cohort than in the DPP placebo arm, likely owing to the substantially longer follow-up in DPPOS. The ML-PR model incorporated HbA1c, FPG, PG2h, HDL-C, and TG as predictive variables. Although these markers are widely recognized indicators of glucose and lipid metabolism, they are also susceptible to lifestyle factors and may exhibit short-term fluctuations. Over an extended follow-up, the predictive power of these baseline measures diminishes as cumulative, unmeasured factors emerge. In addition, DPPOS participants were no longer bound to their original randomized intervention groups, and subsequent lifestyle changes or treatment effects may have altered the natural course of diabetes risk, further contributing to performance decline. These findings underscore that the ML-PR model is more robust for short-term prediction, but may require recalibration and dynamic updating with longitudinal data to remain accurate over longer prediction.

This study identified distinct type 2 diabetes progression risks and intervention responses within prediabetes cohorts, emphasizing the importance of stratified intervention in prediabetes management. ML-PR–based risk stratification also identified substantial differences in treatment efficiency across risk groups. The estimated NNT values were considerably lower in the high-risk group than in the low-risk group, particularly for lifestyle intervention and metformin therapy. These findings suggest that ML-PR may help target preventive interventions to individuals most likely to benefit. Similar to our study, the Prediabetes Lifestyle Intervention Study reported greater benefits from lifestyle intervention in high-risk individuals (22). The use of antihyperglycemic agents to prevent prediabetes progression remains contentious because of limited healthcare resources and competing public health priorities. While major diabetes associations advocate for metformin use in prediabetes, consensus on the appropriate timing and target populations is lacking. The American Diabetes Association recommends considering metformin for high-risk prediabetic individuals, especially those with BMI≥35 kg/m^2^, elevated fasting glucose and HbA1c, or a history of gestational diabetes (23). The Chinese Diabetes Association suggested considering medication for extremely high- and high-risk prediabetes populations after lifestyle intervention (24), though it does not specify a framework for quantifying risk. Emerging evidence reinforces the value of risk stratification. The Beijing Prediabetes Reversion Program study revealed that pioglitazone combined with intensive lifestyle intervention was more effective in preventing diabetes progression in high-risk individuals (9). Likewise, the STOP-Diabetes trial demonstrated that in a prospective setting, intensified pharmacological interventions significantly reduced type 2 diabetes progression in individuals at high risk, although risk stratification was based on traditional clinical factors rather than a predictive risk model (25). Given the potential side effects and costs of medications—such as gastrointestinal discomfort and an increased risk of lactic acidosis—drug use in low-risk individuals should be approached with caution (26, 27).Taken together, we propose that pharmacological treatments and intensified lifestyle interventions could be targeted at high-risk prediabetic individuals through precise risk stratification, while low-risk individuals may initially pursue conventional lifestyle therapy. If patient is self-motivated or had a high expectation of delaying diabetes progression, intensive lifestyle therapy, but not metformin can be considered in the low-risk group due to lower NNT.

Our study demonstrates that a five-variable diabetes progression prediction model effectively predicts not only overall diabetes risk but also stratifies risks for microvascular complications. Subgroup analyses further revealed that the elevated microvascular risk observed in the high-risk group was primarily attributable to significantly higher incidences of nephropathy and retinopathy, whereas no significant difference was noted for neuropathy. This heterogeneity may reflect distinct mechanisms underlying different microvascular complications. Both diabetic nephropathy and retinopathy are strongly driven by chronic hyperglycemia–induced processes, such as microvascular basement membrane thickening, hemodynamic alterations, and oxidative stress accumulation (28, 29). In contrast, the development of neuropathy is influenced by a broader spectrum of factors, including aging, micronutrient deficiencies, and peripheral circulatory impairment (30), which may attenuate its direct sensitivity to short-term variations in glycemic and metabolic parameters. When the model identifies patients at high risk of microvascular complications, clinical evaluation should prioritize renal function monitoring and fundus assessment. Intensive glycemic control, together with optimized lipid and blood pressure management, may help interrupt hyperglycemia-induced microvascular injury, particularly in renal and retinal events. These findings highlight the utility of ML-PR in refining microvascular risk stratification and guiding preventive strategies.

Although the ML-PR model demonstrates robust performance in stratifying type 2 diabetes risk and microvascular events, its predictive utility for cardiovascular outcomes is limited among individuals with prediabetes, largely because traditional vascular risk factors are absent from its inputs. In addition to the risk of developing diabetes, prediabetes is also associated with an increased risk of cardiovascular events, which are generally thought to be attributed to elevated glucose levels and the subsequent onset of type 2 diabetes (5). The prevailing view has emphasized diabetes progression as the main clinical consequence of prediabetes, with prevention strategies focused on delaying this transition to mitigate cardiovascular burden. However, evidence from large cohorts such as the Kailuan cohort suggested that comorbidities, particularly hypertension, contributed substantially to the elevated cardiovascular risk observed in individuals with prediabetes (31, 32). Importantly, most diabetes prevention trials have succeeded in delaying diabetes onset but failed to demonstrate reductions in cardiovascular events (31, 33–35). This discrepancy highlights the need for integrated approaches in prediabetes management, exemplified the emerging cardiovascular–kidney–metabolic syndrome (CKM) framework, which calls for simultaneous management of glycemia and cardiovascular risk factors (36). Within this paradigm, comprehensive care should address both diabetes progression (CKM stage 2) and cardiovascular disease development (CKM stage 4). The ML-PR, driven primarily by glycemic and lipid serum markers, lacks several key CVD risk factors, such as age, sex, smoking status, and blood pressure, explaining its limited discrimination for the incidence of CVD. Our findings therefore support the complementary use of dedicated CVD risk tools, such as PREVENT, for cardiovascular assessment in prediabetic populations. Integrating ML-PR with PREVENT, which incorporates vascular risk predictors, could substantially enhance overall predictive performance and provide a more holistic basis for precision prevention. Although PREVENT successfully identified CVD risk in prediabetic patients, the stratified risk groups did not exhibit differential responses to drug or lifestyle interventions (**Supplementary Figure 10**). Future research should investigate whether prediabetic individuals at high cardiovascular risk could benefit from specific or integrated therapeutic approaches.

Our study has several limitations. First, while DPP and DPPOS provide high-quality data, generalizability may be limited owing to participant demographics, including overrepresentation of overweight and Caucasian individuals. The findings may be less applicable to other racial and ethnic groups, such as African Americans, Hispanics, and other populations, which were underrepresented in the DPP and DPPOS. Second, As these *post hoc* analyses were not prespecified and the original trials were not designed or powered to evaluate risk-stratified or sex-specific interaction effects, the observed ML-PR–intervention interactions should be considered exploratory and interpreted cautiously, given potential residual confounding, bias, and lack of formal adjustment for multiple testing. Prospective risk-stratified intervention studies are needed to confirm whether ML-PR–guided strategies can effectively optimize efficacy, safety, cost, and resource allocation. Third, microvascular events were analyzed via logistic regression due to the lack of time-to-event data. While this approach identifies associations with binary outcomes, it assumes non-time-varying relationships and ignores temporal dynamics, potentially introducing bias from unequal follow-up or competing risks. This may underestimate or overestimate true long-term risks due to differential follow-up times and unaccounted competing risks. Fourth, as the ML-PR model was originally developed for short-term diabetes risk prediction, the long-term risk estimates should be interpreted with appropriate caution, and future studies specifically designed for extended prediction horizons are warranted. Finally, as the DPP and DPPOS cohorts consisted of motivated trial participants undergoing structured interventions and systematic follow-up, which may limit generalizability to real-world prediabetes populations.

In conclusion, the ML-PR model demonstrated robust external validity in the DPP and DPPOS cohorts, effectively identifying individuals at greater risk of type 2 diabetes progression. This study also revealed clear treatment heterogeneity, with high-risk individuals benefiting the most from metformin and lifestyle interventions. These findings underscore the utility of the ML-PR for precision risk stratification and personalized diabetes prevention.

## Data Availability

Publicly available datasets were analyzed in this study. This data can be found here: The DPP and DPP Outcomes Study was conducted by the DPP Research Group and supported by the National Institute of Diabetes and Digestive and Kidney Diseases (NIDDK), the General Clinical Research Center Program (GCRC), the National Institute of Child Health and Human Development (NICHD), the National Institute on Ageing (NIA), the Office of Research on Women’s Health (ORWH), the Office of Research on Minority Health (ORMH), the Centers for Disease Control and Prevention (CDC), and the American Diabetes Association (ADA). The data from the DPP and DPPOS were supplied by the NIDDK Central Repository (NIDDK-CR) and are available for request at https://repository.niddk.nih.gov. This manuscript was not prepared under the auspices of the DPP Research Group and does not represent analyses or conclusions of the DPP Research Group, the CDC, NIDDK-CR, or NIH.
