# Supplementary material for: External validation and application of a machine learning–based model for diabetes progression in prediabetes
Source: Front Endocrinol (Lausanne). 2026 Mar 23;17:1746570. doi: 10.3389/fendo.2026.1746570 (PMC13051266; doi:10.3389/fendo.2026.1746570)
Supplement: Supplementary file 1 [file DataSheet1.docx]

Supplementary Material

# Supplementary Figures and Tables


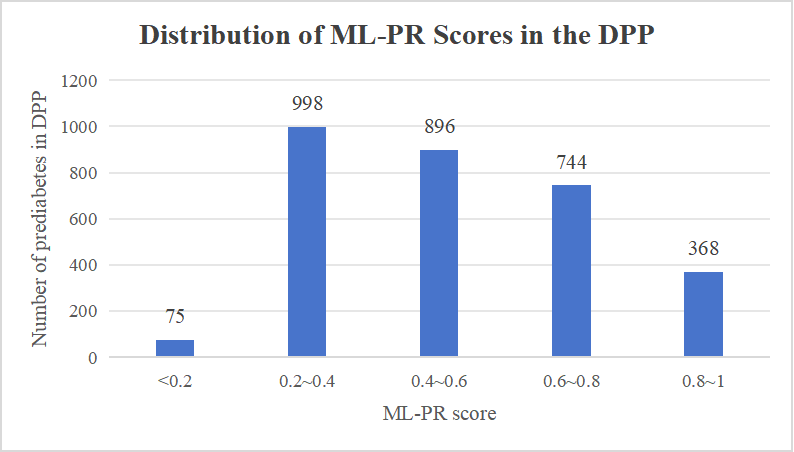


**Supplementary Figure 1****The distribution of ML-PR score by 0.2 increments in the DPP cohort.**

Abbreviations: DPP, Diabetes Prevention Program; ML-PR, machine learning–based model for type 2 diabetes progression


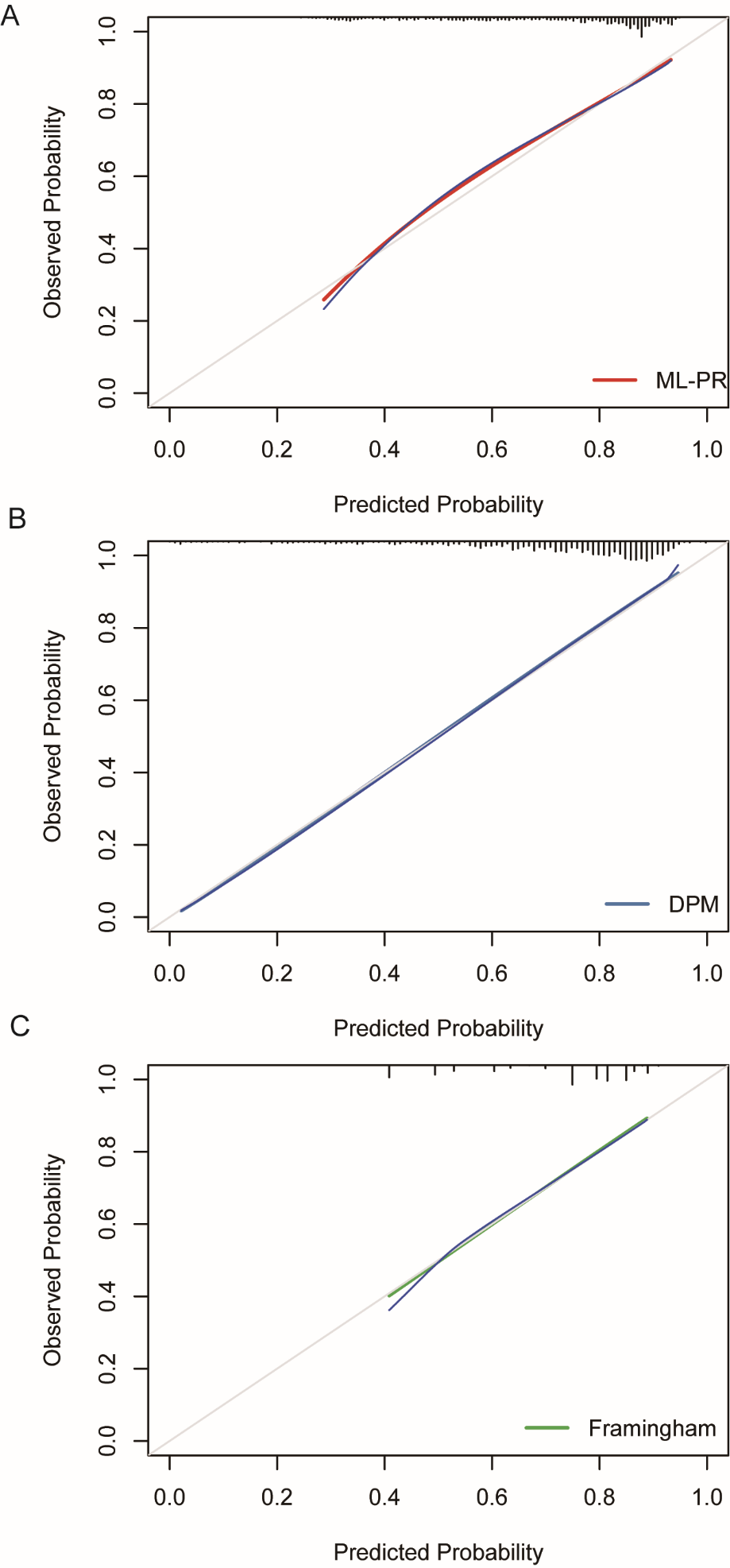


**Supplementary Figure 2. Calibration curves of the type 2 diabetes prediction models for DPP.**

Calibration curves of diabetes prediction models (ML-PR (A), diabetes prediction model (B), and Framingham model(C)) for type 2 diabetes progression in the DPP placebo arm (n=1030).The grey line indicates perfect calibration. The red (ML-PR), blue (DPM) and green (Framingham model) line represents the apparent calibration performance in the placebo arm, while the another blue line denotes the bias-corrected calibration curve obtained through bootstrap resampling.

Abbreviations: DPP, Diabetes Prevention Program; ML-PR, machine learning–based model for type 2 diabetes progression.

**
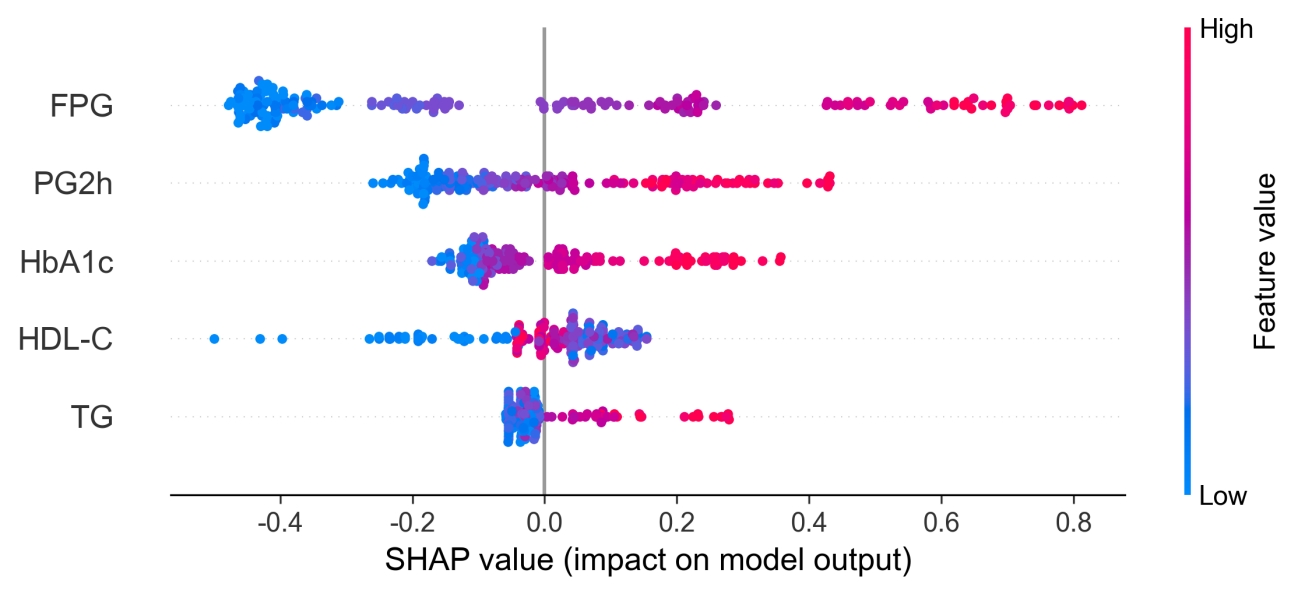
**

**Supplementary Figure 3. The SHapley Additive exPlanations summary plot for the ML-PR model in the external validation cohort.**
The SHapley Additive exPlanations (SHAP) summary plot illustrates the contribution of each predictor—fasting plasma glucose (FPG), 2-hour post-load glucose (PG2h), glycated hemoglobin (HbA1c), high-density lipoprotein cholesterol (HDL-C), and triglycerides (TG)—to the ML-PR risk prediction. Each dot represents an individual participant. The x-axis indicates the SHAP value (impact on model output), where positive values denote increased predicted risk and negative values denote decreased predicted risk. Dot color reflects the feature value (blue = low, red = high).


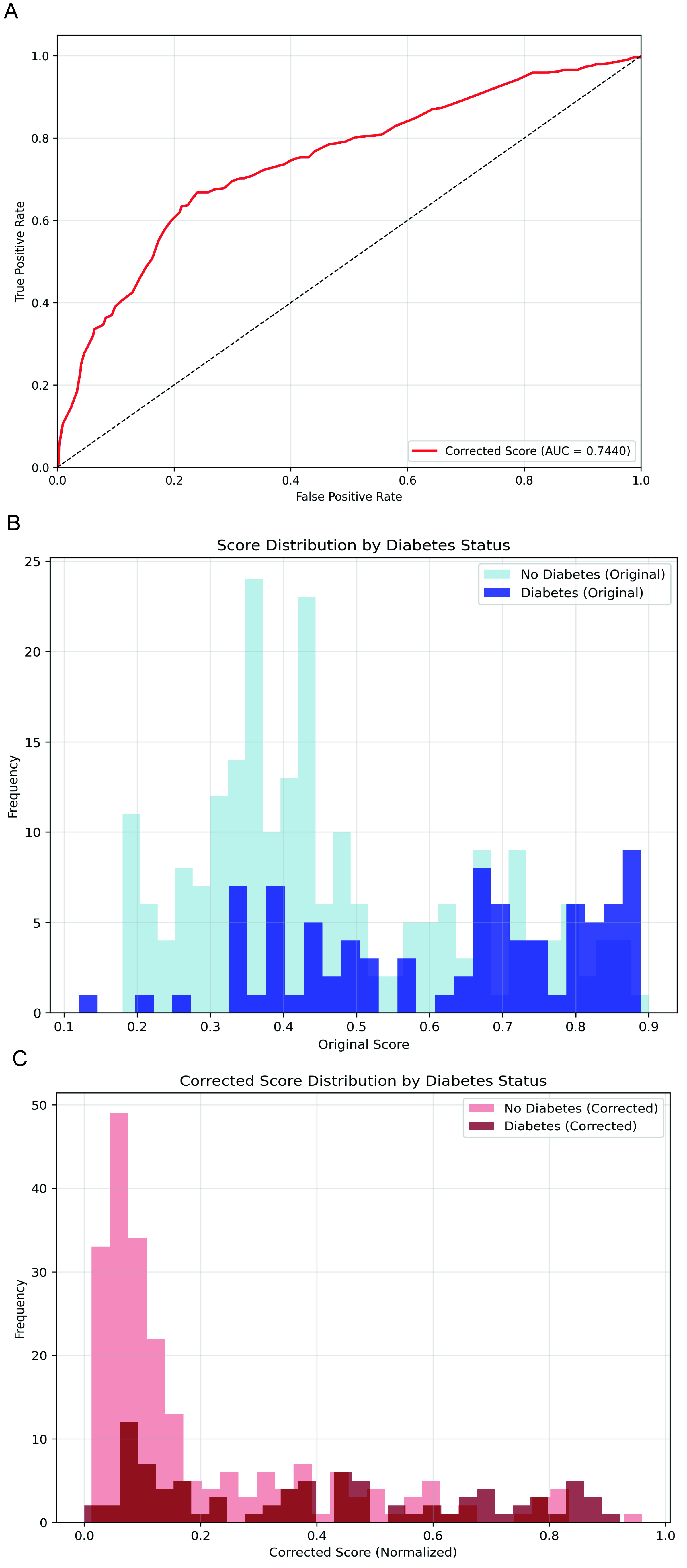


**Supplementary Figure 4. Receiver operating characteristic curves andcalibration curves of the recalibrationmodel for DPP**

Receiver operating characteristic curves for the recalibration model of machine learning-based diabetes progression model (ML-PR)in predicting 3-year type 2 diabetes progression in the DPP placebo arm (A) (n=1024).


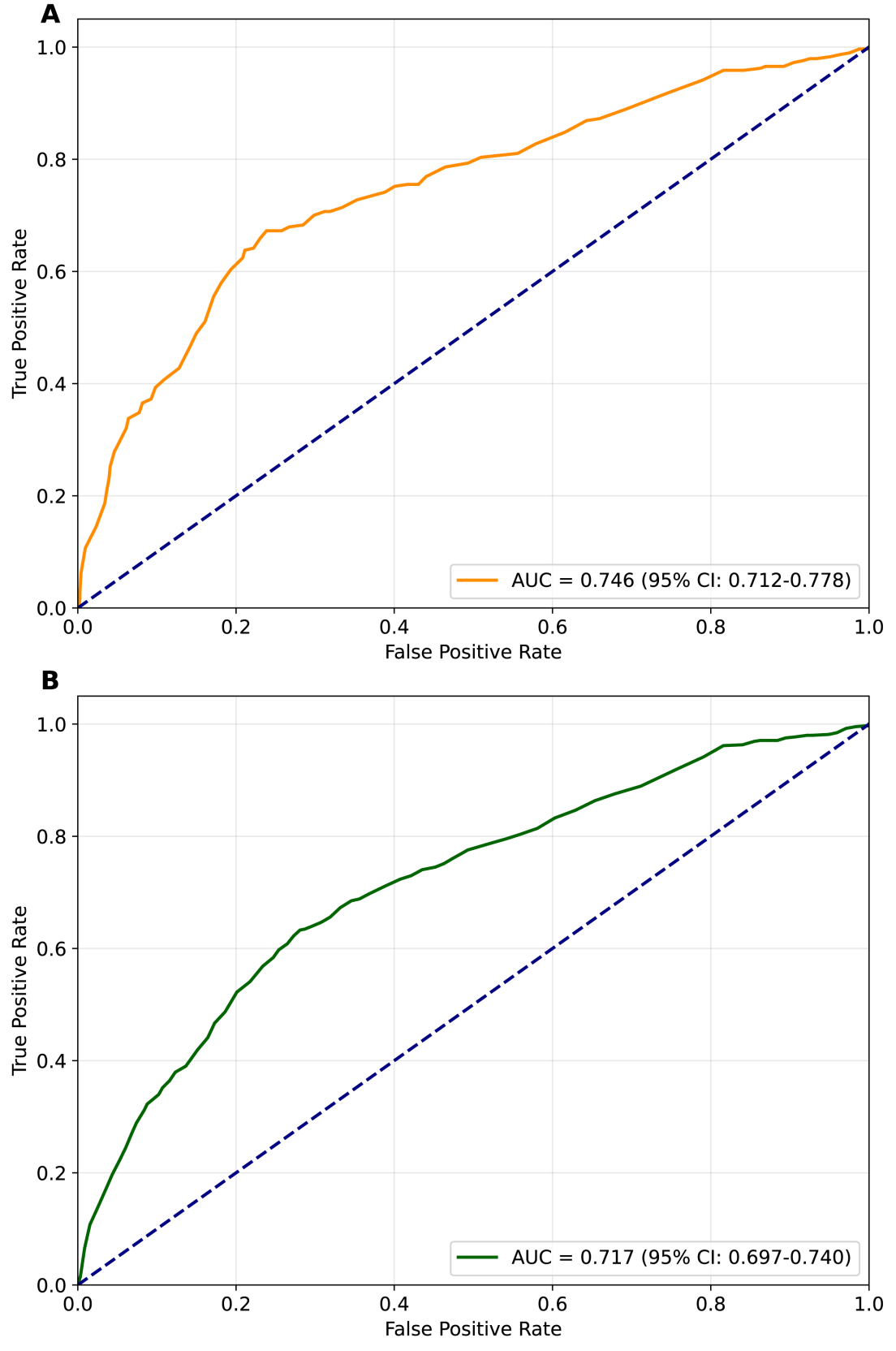


**Supplementary Figure 5. Case-complete analysis of ML-PR for diabetes prediction in DPP.**

Receiver operating characteristic curves for the machine learning-based diabetes progression model (ML-PR)in predicting 3-year type 2 diabetes progression in the DPP placebo arm (A)(n=1024) and total arm(B)(n=3068).

Abbreviations: DPP, Diabetes Prevention Program; ML-PR, machine learning–based model for type 2 diabetes progression.


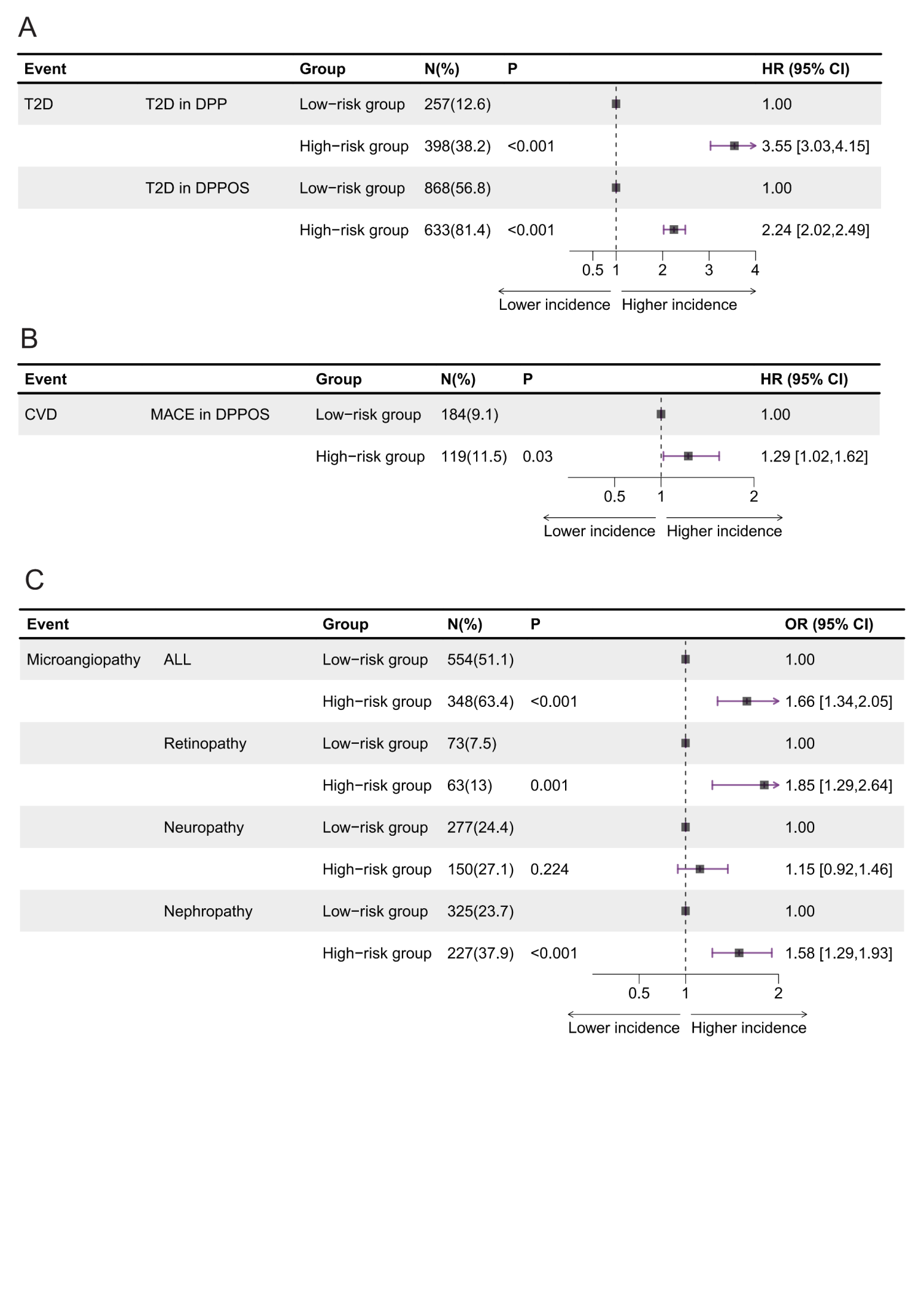


**Supplementary Figure 6. Es****timated risks of type 2 diabetes, cardiovascular disease (CVD), and microvascular outcomes across different risk groups****using the tertile of ML-PR score as the cutoff value.**

HR for incident type 2 diabetes (A)and CVD (B)were calculated viaCox proportional hazards regression, accounting for time-to-event data. ORsformicrovascular outcomes(C) were derived fromlogistic regressiondue to the absence of recorded onset times for these outcomes. All estimates are presented with 95% CIs. The data are summarized as N (%) for categorical representation.

Abbreviations: CVD, cardiovascular disease; CI, confidence interval; DPP, Diabetes Prevention Program; DPPOS, Diabetes Prevention Program Outcome Study; HR, hazard ratio; MACE, major adverse cardiovascular events; OR, odds ratio;T2D, type 2 diabetes.


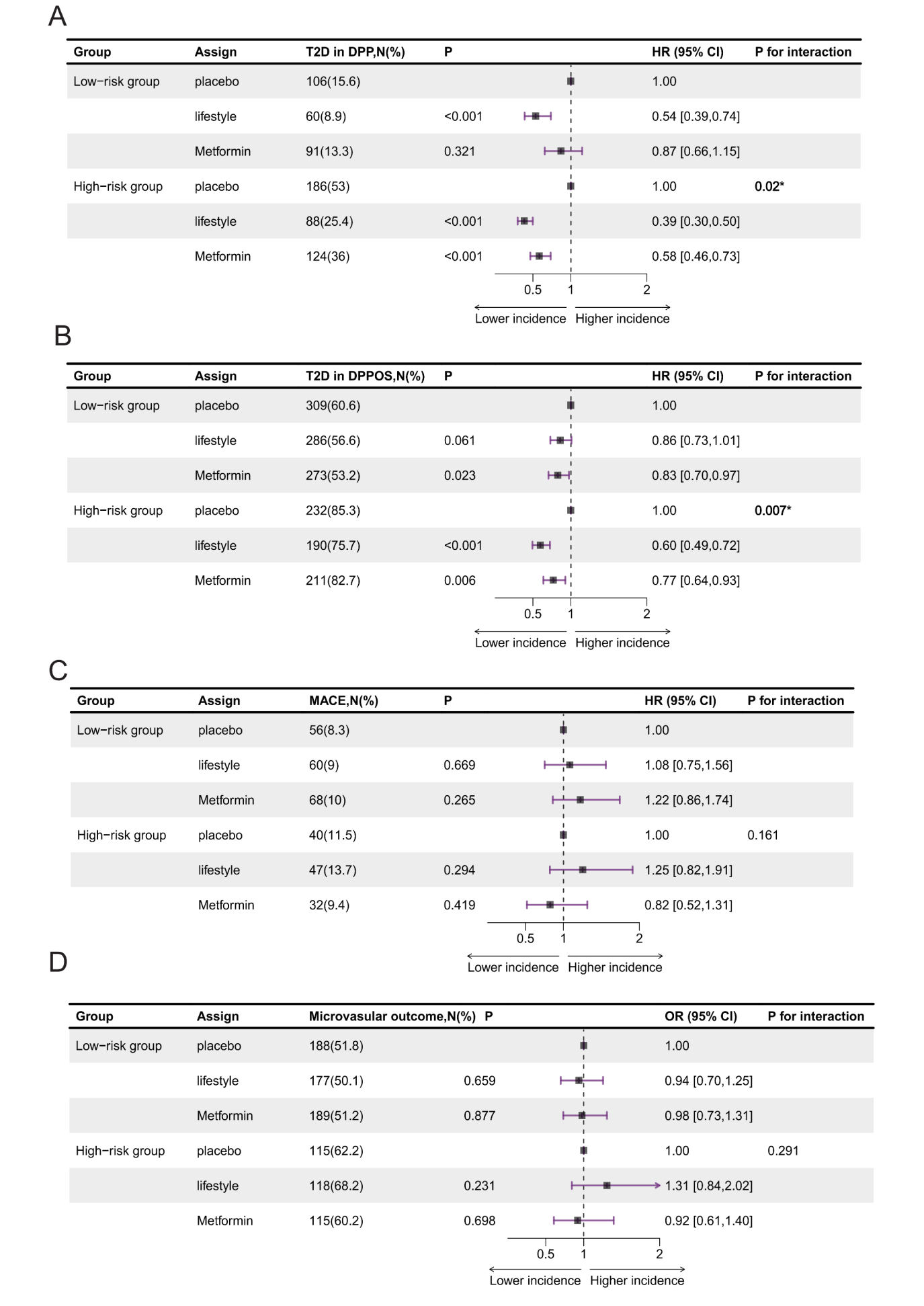


**Supplementary Figure 7. Type 2 diabetes progression, cardiovascular events and microvascular outcomes under interventions across risk groups using the tertile of ML-PR score as the cutoff value.**

HR in reference to placebo intervention for progression to type 2 diabetes in the high- and low-risk groups at the end of DPP(A) and DPPOS(B). HR in reference to placebo intervention for progression to CVD in high- and low-risk groups at the end of DPPOS (C). OR in reference to placebo intervention for progression to microvascular outcomes in the high- and low-risk groups at the end of DPPOS (D). HR for type 2 diabetes and CVD events were analysed via Cox regression, and OR for microvascular outcomes was analysed via logistic regression, as the exact time of onset for microvascular outcomes was not recorded. The P value for interaction was assessed via the Wald test. All estimates are presented with 95% CIs. The data are summarized as N (%) for categorical representation.

Abbreviations: CVD, cardiovascular disease; CI, confidence interval; DPP, Diabetes Prevention Program; DPPOS, Diabetes Prevention Program Outcome Study; HR, hazard ratio; MACE, major adverse cardiovascular events; OR, odds ratio; T2D, type 2 diabetes.


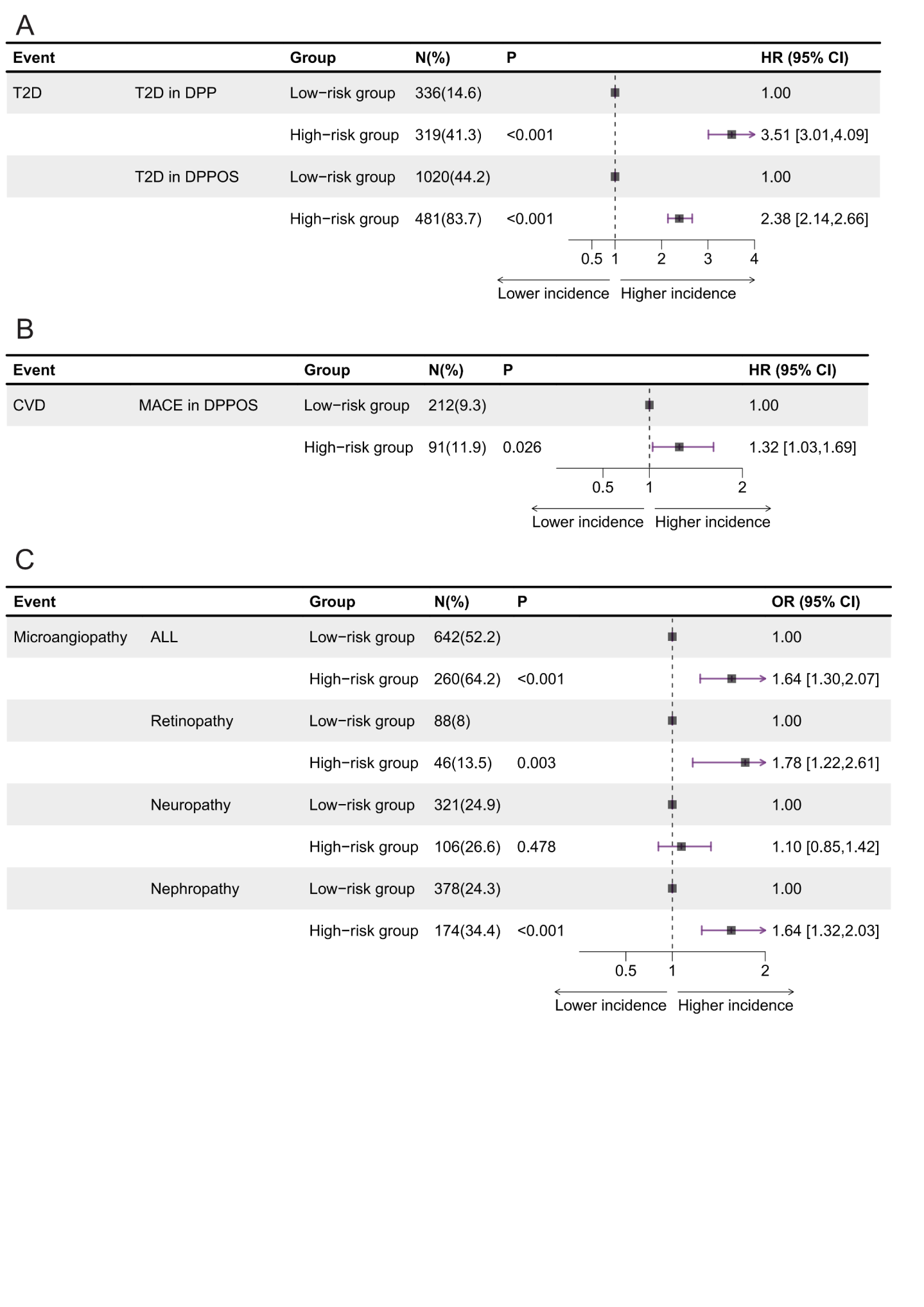


**Supplementary Figure 8. Estimated risks of type 2 diabetes, cardiovascular disease (CVD), and microvascular outcomes across different risk groups using the quartile of ML-PR score as the cutoff value.**

HR for incident type 2 diabetes (A) and CVD (B) were calculated via Cox proportional hazards regression, accounting for time-to-event data. ORs for microvascular outcomes(C) were derived from logistic regression due to the absence of recorded onset times for these outcomes. All estimates are presented with 95% CIs. The data are summarized as N (%) for categorical representation.

Abbreviations: CVD, cardiovascular disease; CI, confidence interval; DPP, Diabetes Prevention Program; DPPOS, Diabetes Prevention Program Outcome Study; HR, hazard ratio; MACE, major adverse cardiovascular events; OR, odds ratio;T2D, type 2 diabetes.


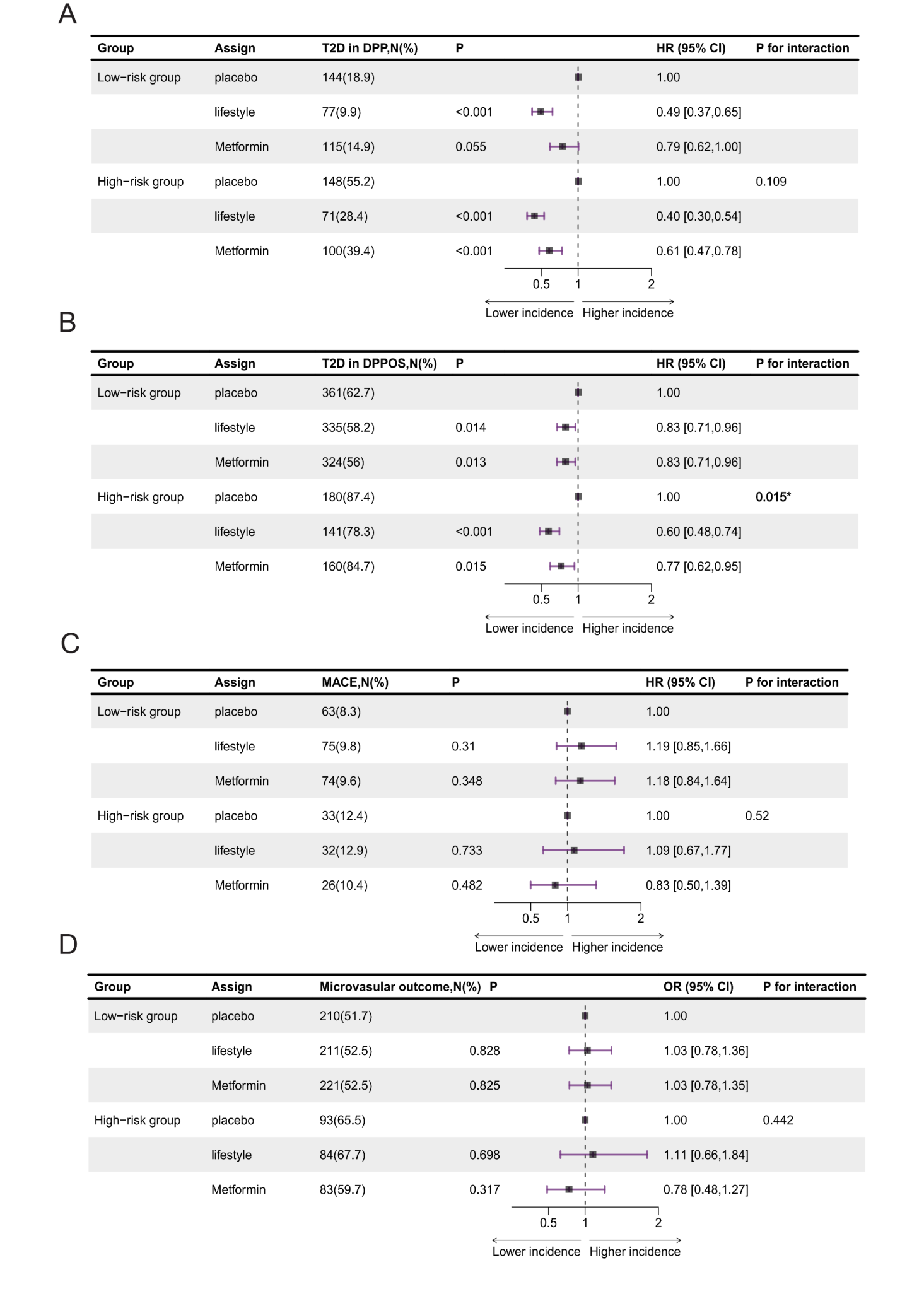


**Supplementary Figure 9. Type 2 diabetes progression, cardiovascular events and microvascular outcomes under interventions across risk groups using the quartile of ML-PR score as the cutoff value.**

HR in reference to placebo intervention for progression to type 2 diabetes in the high- and low-risk groups at the end of DPP(A) and DPPOS(B). HR in reference to placebo intervention for progression to CVD in high- and low-risk groups at the end of DPPOS (C). OR in reference to placebo intervention for progression to microvascular outcomes in the high- and low-risk groups at the end of DPPOS (D). HR for type 2 diabetes and CVD events were analysed via Cox regression, and OR for microvascular outcomes was analysed via logistic regression, as the exact time of onset for microvascular outcomes was not recorded. The P value for interaction was assessed via the Wald test. All estimates are presented with 95% CIs. The data are summarized as N (%) for categorical representation.

Abbreviations: CVD, cardiovascular disease; CI, confidence interval; DPP, Diabetes Prevention Program; DPPOS, Diabetes Prevention Program Outcome Study; HR, hazard ratio; MACE, major adverse cardiovascular events; OR, odds ratio; T2D, type 2 diabetes.
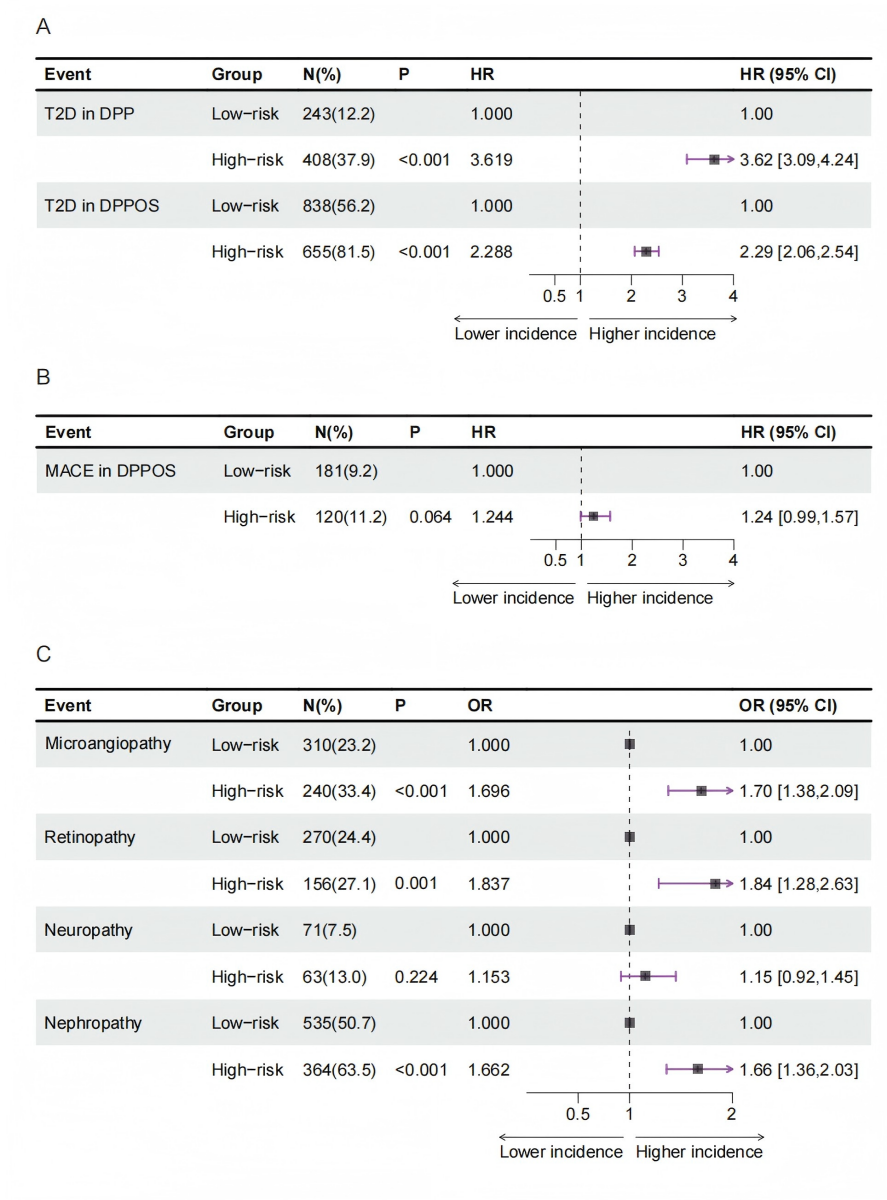

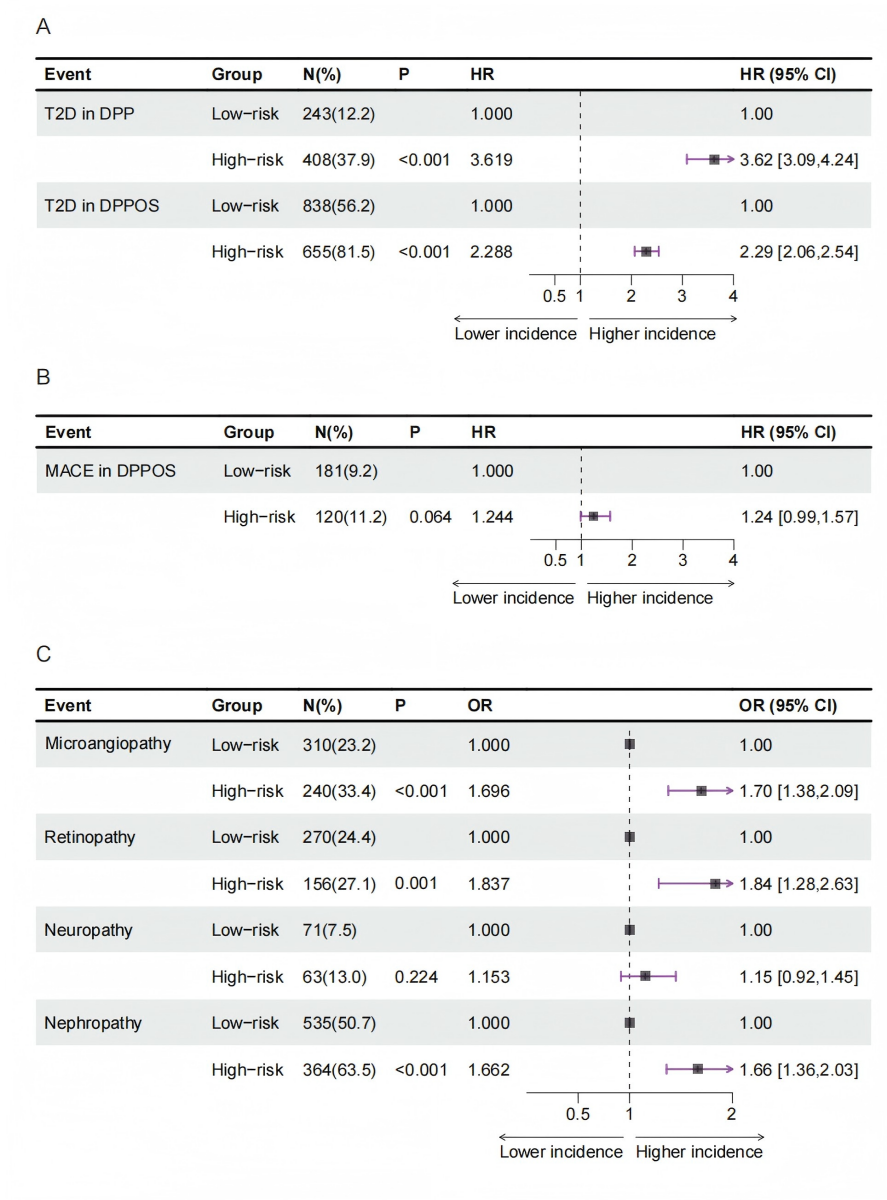


**Supplementary Figure 10. Estimated risks of type 2 diabetes, cardiovascular disease (CVD), and microvascular outcomes across different risk groups using Youden index–derived cutoff.**

HR for incident type 2 diabetes (A) and CVD (B) were calculated via Cox proportional hazards regression, accounting for time-to-event data. ORs for microvascular outcomes(C) were derived from logistic regression due to the absence of recorded onset times for these outcomes. All estimates are presented with 95% CIs. The data are summarized as N (%) for categorical representation. Abbreviations: CVD, cardiovascular disease; CI, confidence interval; DPP, Diabetes Prevention Program; DPPOS, Diabetes Prevention Program Outcome Study; HR, hazard ratio; MACE, major adverse cardiovascular events; OR, odds ratio; T2D, type 2 diabetes.


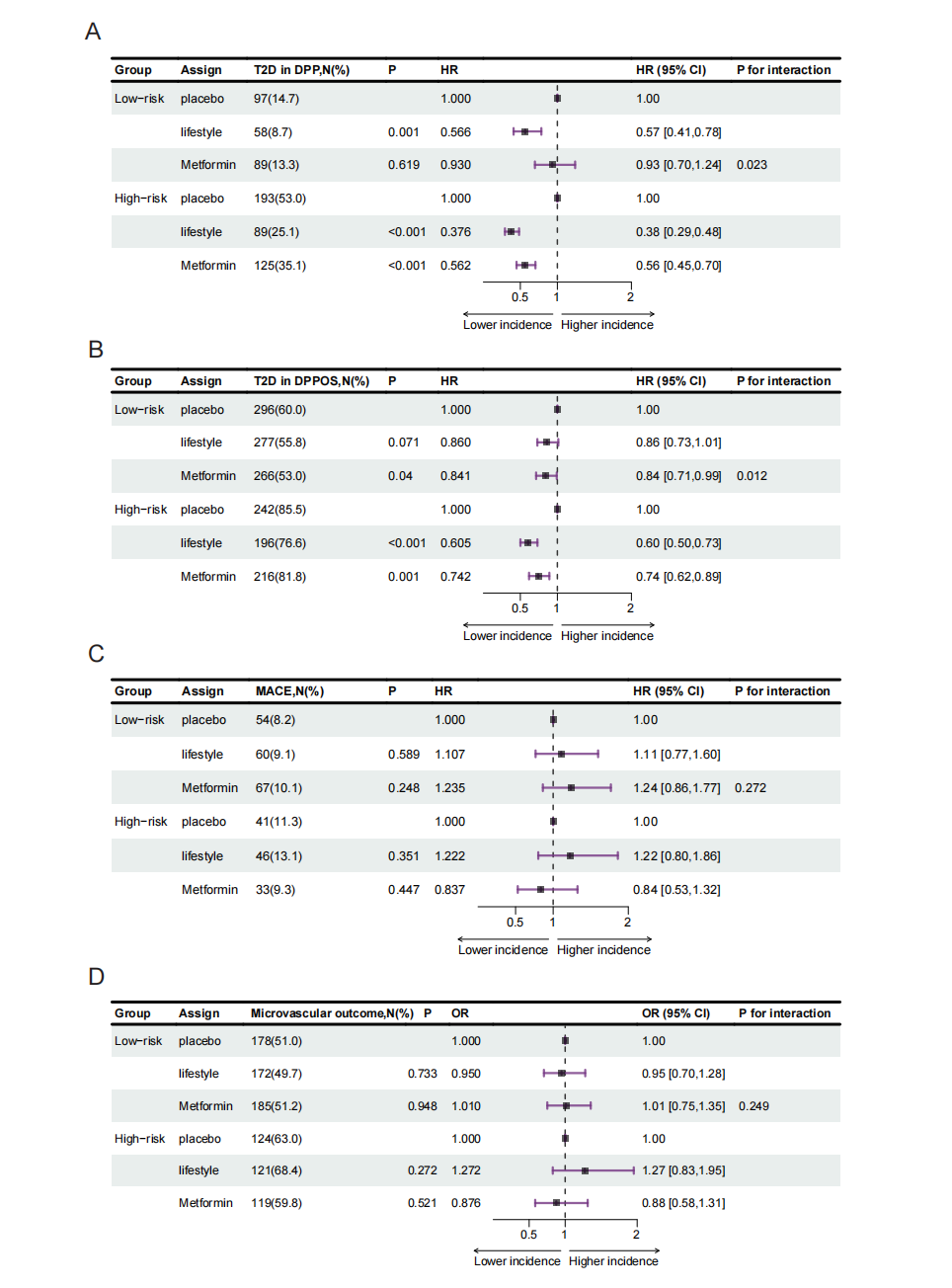


**Supplementary Figure 11. Type 2 diabetes progression, cardiovascular events and microvascular outcomes under interventions across risk groups Youden index–derived cutoff**

HR in reference to placebo intervention for progression to type 2 diabetes in the high- and low-risk groups at the end of DPP(A) and DPPOS(B). HR in reference to placebo intervention for progression to CVD in high- and low-risk groups at the end of DPPOS (C). OR in reference to placebo intervention for progression to microvascular outcomes in the high- and low-risk groups at the end of DPPOS (D). HR for type 2 diabetes and CVD events were analysed via Cox regression, and OR for microvascular outcomes was analysed via logistic regression, as the exact time of onset for microvascular outcomes was not recorded. The P value for interaction was assessed via the Wald test. All estimates are presented with 95% CIs. The data are summarized as N (%) for categorical representation. Abbreviations: CVD, cardiovascular disease; CI, confidence interval; DPP, Diabetes Prevention Program; DPPOS, Diabetes Prevention Program Outcome Study; HR, hazard ratio; MACE, major adverse cardiovascular events; OR, odds ratio; T2D, type 2 diabetes.

**
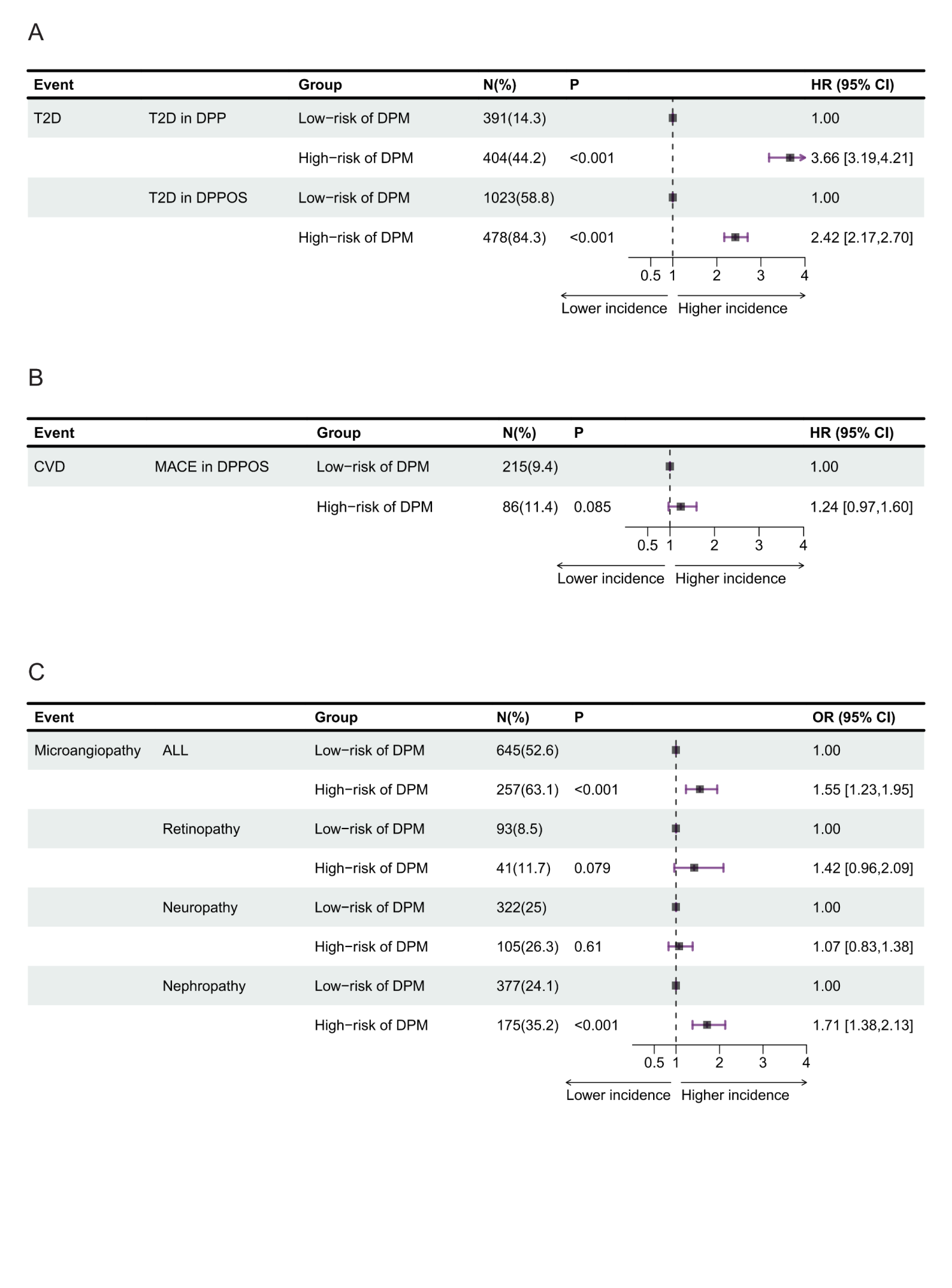
**

**Supplementary Figure 12. Estimated risks of type 2 diabetes, cardiovascular disease (CVD), and microvascular outcomes across risk groups stratified by the diabetes prediction model (DPM)**

HR for incident type 2 diabetes (A) and CVD (B) were calculated via Cox proportional hazards regression, accounting for time-to-event data. ORs for microvascular outcomes (C) were derived from logistic regression due to the absence of recorded onset times for these outcomes. All estimates are presented with 95% CIs. The data are summarized as N (%) for categorical representation.

Abbreviations: CVD, cardiovascular disease; CI, confidence interval; DPM, Diabetes Prediction Model;DPP, Diabetes Prevention Program; DPPOS, Diabetes Prevention Program Outcome Study; HR, hazard ratio;MACE, major adverse cardiovascular events; OR, odds ratio;T2D, type 2 diabetes.

**
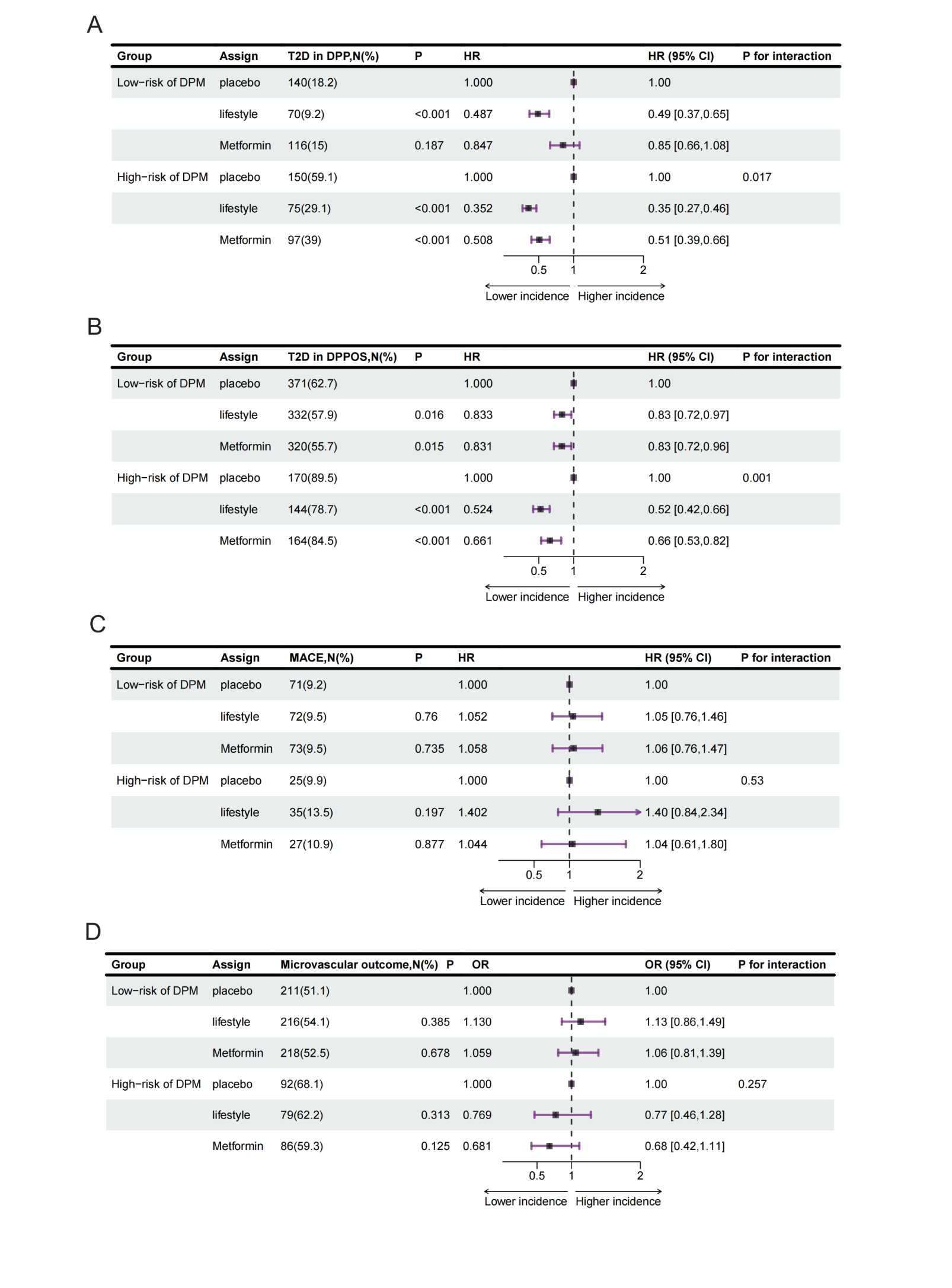
**

**Supplementary Figure 13. Type 2 diabetes progression and cardiovascular and microvascular outcomes under intervention among risk groups according to the diabetes prediction model(DPM).**

HR in reference to placebo intervention for progression to type 2 diabetes in the high- and low-risk groups at the end of DPP(A) and DPPOS(B). HR in reference to placebo intervention for progression to CVD in high- and low-risk groups at the end of DPPOS (C). OR in reference to placebo intervention for progression to microvascular outcomes in the high- and low-risk groups at the end of DPPOS (D). HR for type 2 diabetes and CVD events were analysed via Cox regression, and OR for microvascular outcomes was analysed via logistic regression, as the exact time of onset for microvascular outcomes was not recorded. The P value for interaction was assessed via the Wald test. All estimates are presented with 95% CIs. The data are summarized as N (%) for categorical representation.

Abbreviations: CVD, cardiovascular disease; CI, confidence interval; DPM, Diabetes Prediction Model; DPP, Diabetes Prevention Program; DPPOS, Diabetes Prevention Program Outcome Study; HR, hazard ratio; MACE, major adverse cardiovascular events; OR, odds ratio; T2D, type 2 diabetes.


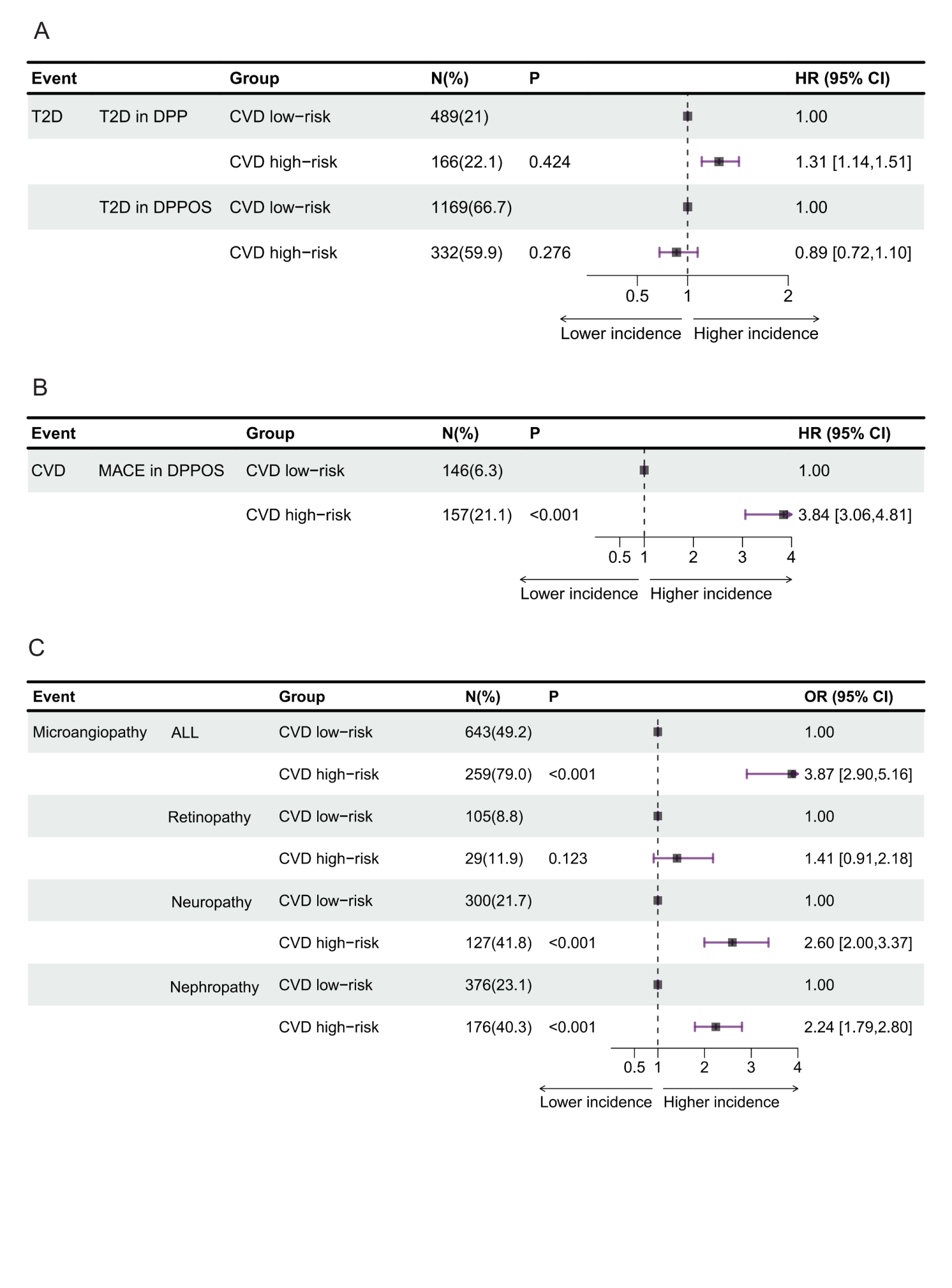


**Supplementary Figure 14. Estimated risk for type 2 diabetes, cardiovascular disease (CVD), and microvascular outcomes across CVD risk groups.**

HR for incident type 2 diabetes (A) and CVD (B) were calculated via Cox proportional hazards regression, accounting for time-to-event data. ORs for microvascular outcomes (C) were derived from logistic regression due to the absence of recorded onset times for these outcomes. All estimates are presented with 95% CIs. The data are summarized as N (%) for categorical representation.

Abbreviations: CVD, cardiovascular disease; CI, confidence interval; DPP, Diabetes Prevention Program; DPPOS, Diabetes Prevention Program Outcome Study; HR, hazard ratio; MACE, major adverse cardiovascular events; OR, odds ratio; T2D, type 2 diabetes.

**
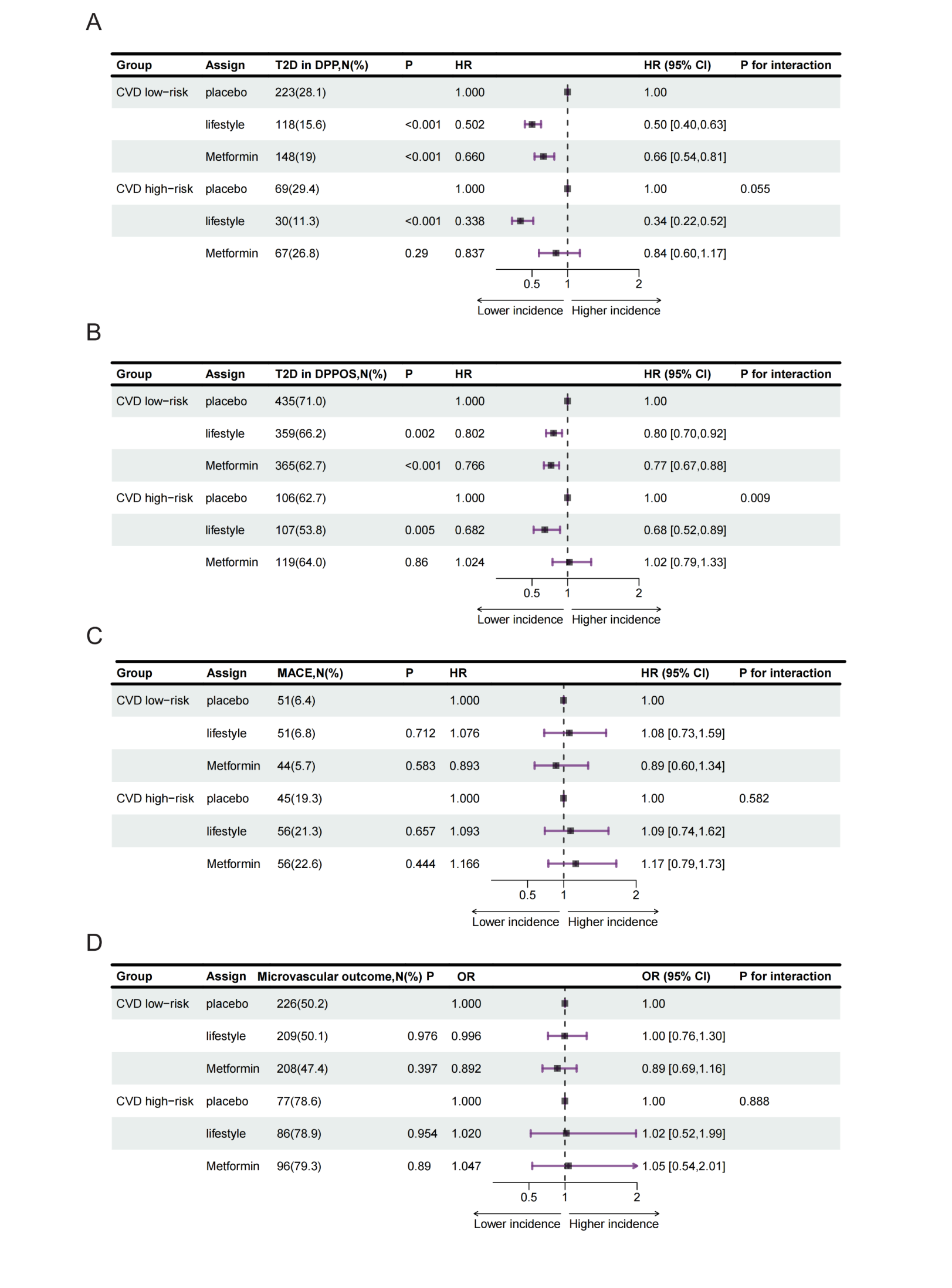
**

**Supplementary Figure 15. Progression of type 2 diabetes, cardiovascular events, and microvascular outcomes under interventions across CVD risk groups**

HR in reference to placebo intervention for progression to type 2 diabetes in the high- and low-risk groups at the end of DPP(A) and DPPOS(B). HR in reference to placebo intervention for progression to CVD in high- and low-risk groups at the end of DPPOS (C). OR in reference to placebo intervention for progression to microvascular outcomes in the high- and low-risk groups at the end of DPPOS (D). HR for type 2 diabetes and CVD events were analysed via Cox regression, and OR for microvascular outcomes was analysed via logistic regression, as the exact time of onset for microvascular outcomes was not recorded. The P value for interaction was assessed via the Wald test. All estimates are presented with 95% CIs. The data are summarized as N (%) for categorical representation.

Abbreviations: CVD, cardiovascular disease; CI, confidence interval; DPP, Diabetes Prevention Program; DPPOS, Diabetes Prevention Program Outcome Study; HR, hazard ratio; MACE, major adverse cardiovascular events; OR, odds ratio; T2D, type 2 diabetes.


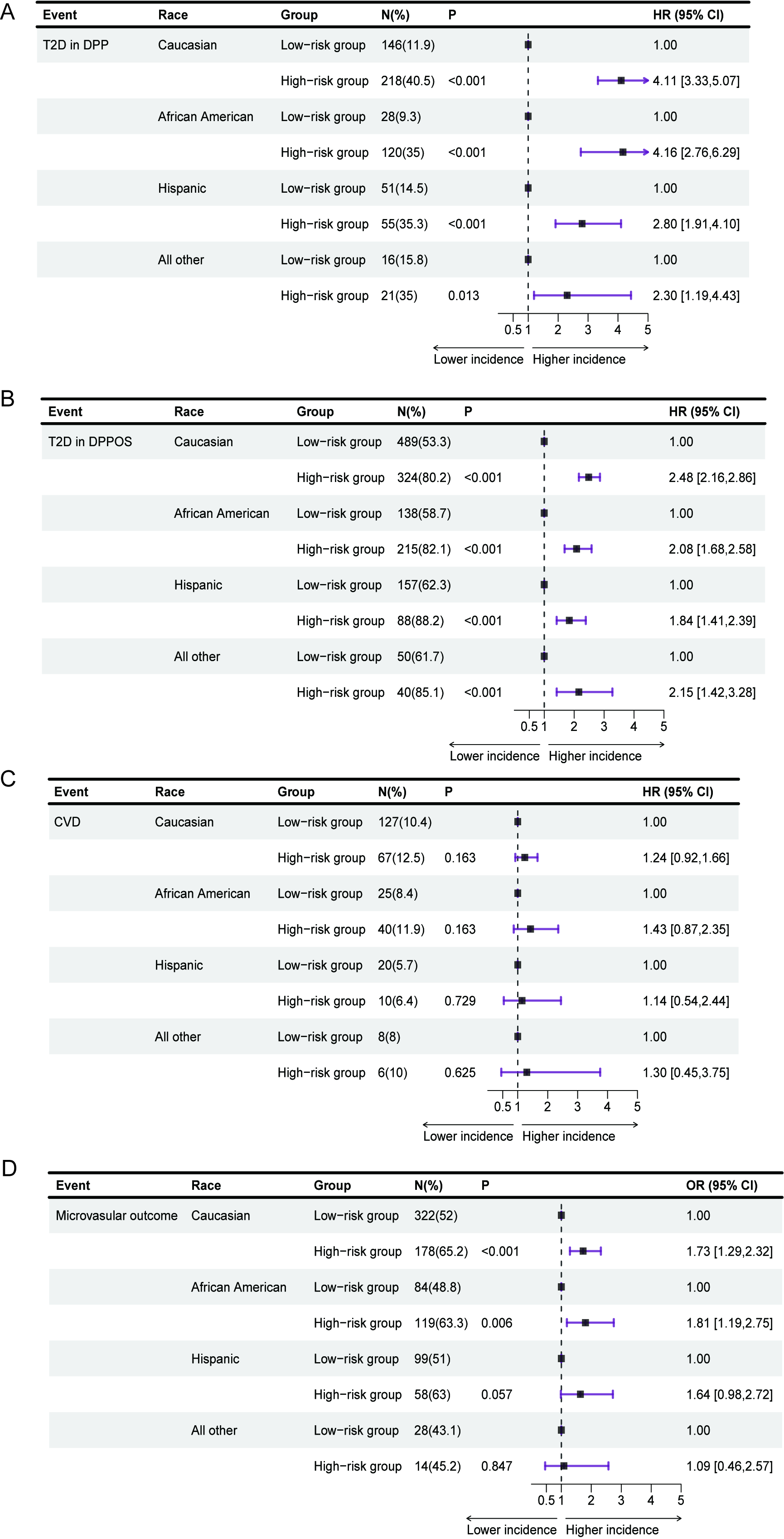


**Supplementary Figure 16. Estimated risks of type 2 diabetes, cardiovascular disease (CVD),and outcomes across risk groups stratified by ML-PR in different races.**

HR for incident type 2 diabetes (A) and CVD (B) were calculated via Cox proportional hazards regression, accounting for time-to-event data in different races. ORs for microvascular outcomes (C) were derived from logistic regression due to the absence of recorded onset times for these outcomes. All estimates are presented with 95% CIs. The data are summarized as N (%) for categorical representation.

Abbreviations: CVD, cardiovascular disease; CI, confidence interval; DPM, Diabetes Prediction Model;DPP, Diabetes Prevention Program; DPPOS, Diabetes Prevention Program Outcome Study; HR, hazard ratio;MACE, major adverse cardiovascular events; OR, odds ratio;T2D, type 2 diabetes.

|  | **ML-PR** | **DPM** | **Framingham model** |
| --- | --- | --- | --- |
| Cut-off value | 0.60 | Upper quarter value | 12 |
| ROC AUC in DPP placebo arm (n=1030) | 0.74  (95%C1,0.71-0.78) | 0.76  (95%C1,0.73-0.80) | 0.71  (95%C1,0.67-0.74) |
| ROC AUC in DPP total cohort (n=3081) | 0.72  (95%C1,0.70-0.74) | 0.74  (95%Cl, 0.71-0.76) | 0.67  (95%C1,0.65-0.70) |
| ROC AUC in DPPOS placebo arm (n=782) | 0.69  (95%C1,0.64-0.72) | 0.70  (95%C1,0.67-0.74) | 0.67  (95%C1,0.64-0.71) |
| ROC AUC in DPPOS total arm (n=2306) | 0.67  (95%C1,0.65-0.70) | 0.69  (95%C1,0.67-0.71) | 0.65  (95%C1,0.63-0.68) |
| Accuracy | 0.71 | 0.75 | 0.71 |
| Sensitivity | 0.64 | 0.51 | 0.50 |
| Specificity | 0.72 | 0.82 | 0.82 |
| Positive Predictive Value | 0.39 | 0.44 | 0.59 |
| Negative Predictive Value | 0.88 | 0.86 | 0.76 |
| F_1_ Score | 0.49 | 0.47 | 0.27 |

**Supplementary Table 1 Model performance of diabetes prediction models for DPP and DPPOS**

Abbreviations: CI, confidence interval; ROC AUC, area under the curve; DPP, Diabetes Prevention Program; DPPOS, Diabetes Prevention Program Outcome Study;DPM, diabetes prediction model; ML-PR, machine learning–based model for type 2 diabetes progression.·

|  | **Caucasian** | **African American** | **Hispanic** | **All other** |
| --- | --- | --- | --- | --- |
| N in DPP | 1768 | 644 | 508 | 161 |
| ROC AUC in DPP placebo arm (n=1030) | 0.77  (95%C1,0.73-0.82) | 0.70  (95%C1,0.63-0.78) | 0.72  (95%C1,0.65-0.81) | 0.65  (95%C1,0.50-0.81) |
| ROC AUC in DPP total cohort (n=3081) | 0.74  (95%C1,0.71-0.74) | 0.71  (95%Cl,0.67-0.76) | 0.69  (95%C1,0.63-0.75) | 0.64  (95%C1,0.53-0.75) |
| N in DPPOS | 1322 | 497 | 359 | 128 |
| ROC AUC in DPPOS placebo arm (n=782) | 0.69  (95%C1,0.64-0.74) | 0.70  (95%C1,0.61-0.78) | 0.62  (95%C1,0.51-0.72) | 0.72  (95%C1,0.57-0.87) |
| ROC AUC in DPPOS total arm (n=2306) | 0.68  (95%C1,0.65-0.71) | 0.67  (95%C1,0.62-0.72) | 0.61  (95%C1,0.55-0.73) | 0.70  (95%C1,0.61-0.80) |
| Accuracy | 0.73 | 0.60 | 0.70 | 0.65 |
| Sensitivity | 0.60 | 0.81 | 0.52 | 0.56 |
| Specificity | 0.77 | 0.54 | 0.74 | 0.67 |
| Positive Predictive Value | 0.40 | 0.34 | 0.35 | 0.34 |
| Negative Predictive Value | 0.88 | 0.90 | 0.86 | 0.84 |
| F_1_ Score | 0.48 | 0.48 | 0.42 | 0.43 |

**Supplementary Table 2 Model performance of diabetes prediction models for DPP and DPPOS in different racial groups**

Abbreviations: CI, confidence interval;ROC AUC, area under the curve; DPP, Diabetes Prevention Program;DPPOS, Diabetes Prevention Program Outcome Study.

| Variable | Total N | Missing N | Missing (%) |
| --- | --- | --- | --- |
| FPG (mmol/L) | 3081 | 0 | 0 |
| PG2h (mmol/L) | 3081 | 0 | 0 |
| HbA1c (%) | 3073 | 8 | 0.25 |
| HDL-C (mmol/L) | 3076 | 5 | 0.16 |
| TG (mmol/L) | 3076 | 5 | 0.16 |

**Supplement Table 3. Missing data summary for predictors included in the ML-PR model.**

This table presents the total sample size, number of missing observations, and percentage of missingness for each predictor variable used in the ML-PR model in the external validation cohort.
